# Tryptophan plays an important role in yeast’s tolerance to isobutanol

**DOI:** 10.1186/s13068-021-02048-z

**Published:** 2021-10-13

**Authors:** Hsien-Lin Liu, Christine H.-T. Wang, En-Pei Isabel Chiang, Chieh-Chen Huang, Wen-Hsiung Li

**Affiliations:** 1 Program in Microbial Genomics, National Chung Hsing University and Academia Sinica, Taipei, Taiwan; 2 Biodiversity Research Center, Academia Sinica, 128 Academia Road, Sec. 2, Taipei, 115 Taiwan; 3 Department of Life Sciences, National Chung Hsing University, No. 145, Xingda Rd., Taichung, 402 Taiwan; 4 Department of Food Science and Biotechnology, National Chung Hsing University, No. 145, Xingda Rd., Taichung, 402 Taiwan; 5 Department of Ecology and Evolution, University of Chicago, Chicago, IL 60637 USA; 6 Innovation and Development Center of Sustainable Agriculture, National Chung Hsing University, No. 145, Xingda Rd. , Taichung, 402 Taiwan

**Keywords:** Isobutanol, Tryptophan, Gene-deletion library screening, Transcriptomic analysis

## Abstract

**Background:**

Isobutanol is considered a potential biofuel, thanks to its high-energy content and octane value, limited water solubility, and compatibility with gasoline. As its biosynthesis pathway is known, a microorganism, such as *Saccharomyces cerevisiae*, that inherently produces isobutanol, can serve as a good engineering host. Isobutanol’s toxicity, however, is a major obstacle for bioproduction. This study is to understand how yeast tolerates isobutanol.

**Results:**

A *S. cerevisiae* gene-deletion library with 5006 mutants was used to screen genes related to isobutanol tolerance. Image recognition was efficiently used for high-throughput screening via colony size on solid media. In enrichment analysis of the 161 isobutanol-sensitive clones identified, more genes than expected were mapped to tryptophan biosynthesis, ubiquitination, and the pentose phosphate pathway (PPP). Interestingly, adding exogenous tryptophan enabled both tryptophan biosynthesis and PPP mutant strains to overcome the stress. In transcriptomic analysis, cluster analysis of differentially expressed genes revealed the relationship between tryptophan and isobutanol stress through some specific cellular functions, such as biosynthesis and transportation of amino acids, PPP, tryptophan metabolism, nicotinate/nicotinamide metabolism (e.g., nicotinamide adenine dinucleotide biosynthesis), and fatty acid metabolism.

**Conclusions:**

The importance of tryptophan in yeast’s tolerance to isobutanol was confirmed by the recovery of isobutanol tolerance in defective strains by adding exogenous tryptophan to the growth medium. Transcriptomic analysis showed that amino acid biosynthesis- and transportation-related genes in a tryptophan biosynthesis-defective host were up-regulated under conditions similar to nitrogen starvation. This may explain why ubiquitination was required for the protein turnover. PPP metabolites may serve as precursors and cofactors in tryptophan biosynthesis to enhance isobutanol tolerance. Furthermore, the tolerance mechanism may also be linked to tryptophan downstream metabolism, including the kynurenine pathway and nicotinamide adenine dinucleotide biosynthesis. Both pathways are responsible for cellular redox balance and anti-oxidative ability. Our study highlights the central role of tryptophan in yeast’s isobutanol tolerance and offers new clues for engineering a yeast host with strong isobutanol tolerance.

**Supplementary Information:**

The online version contains supplementary material available at 10.1186/s13068-021-02048-z.

## Background

Using renewable feedstock to produce energy can reduce the usage of petroleum. Indeed, bio-alcohols are potential gasoline replacements. Among them, isobutanol, a branched-chain alcohol, is superior to ethanol in properties such as a higher energy content, limited solubility in water and no stress cracking in pipelines. In addition, isobutanol has a higher octane number than the linear *n*-butanol and it allows blending with gasoline in a greater amount due to its lower oxygen content than that of ethanol. Moreover, its gasoline compatibility allows the use of existing infrastructure and combustion engines [1, 2].

For bio-based production of isobutanol, *Saccharomyces cerevisiae* has the advantages of tolerance to harsh fermentation environment, immunity to phage contamination, and simple separation. Furthermore, as *S. cerevisiae* has been commonly used in the current bio-ethanol production, the same host organism facilitates a simple transition to isobutanol production with existing fermentation facilities. *S. cerevisiae* inherently produces isobutanol through the valine Ehrlich degradation pathway. Moreover, the well-established genetic tools in *S. cerevisiae* and modern synthetic biology techniques allow for the engineering of yeast hosts for bioproduction of chemicals. Many efforts have been made toward yeast engineering for isobutanol production [3–5]. However, as the production of a novel compound creates a stress to the host, it becomes a hurdle in mass production [6]. To solve this problem, genetic modifications have been made to obtain tolerant strains [7, 8]. Our previous study has provided an example for increasing ethanol and *n*-butanol productivity through enhanced tolerance of the host cell by removing oxidative stress in *E. coli* [9]. As isobutanol toxicity is a major constraint on bioproduction, understanding and improving microbial tolerance to isobutanol has become a hot topic [10]. Kuroda et al. [10] screened a gene-deletion library and discovered the importance of the pentose phosphate pathway (PPP) and tryptophan biosynthesis for yeast isobutanol tolerance. Moreover, deletion of GLN3, a transcriptional activator nitrogen catabolite repression system, was found to increase isobutanol tolerance by blocking the nitrogen starvation signal induced by isobutanol, and the yield of isobutanol was therefore increased [10].

To further understand the mechanism of yeast isobutanol tolerance, we employed a gene-deletion library with 5006 mutants of *S. cerevisiae* (YKO MATa Strain Collection-Glycerol Stocks, Catalog #: YSC1053) and solid-based colony image screening to identify yeast genes and metabolic pathways related to isobutanol tolerance. Enrichment analysis was then employed to categorize the isobutanol-sensitive genes in terms of their functions, and tryptophan biosynthesis, ubiquitination, and the pentose phosphate pathway (PPP) were found to be involved in isobutanol tolerance. To test the importance of tryptophan biosynthesis, we studied whether exogenous tryptophan can recover isobutanol tolerance of tryptophan biosynthesis-mutant strains. Finally, transcriptomic analysis was conducted to investigate possible molecular mechanisms underlining yeast’s isobutanol tolerance. Cluster analysis of differentially expressed genes (DEGs) was applied to infer the correlation between cellular responses and to map the relationship between tryptophan and isobutanol stress onto specific functions. Our study offers an integrated view for the role of tryptophan in yeast’s isobutanol tolerance.

## Results

### Screening for genes and pathways related to isobutanol tolerance

**Fig. 1:**
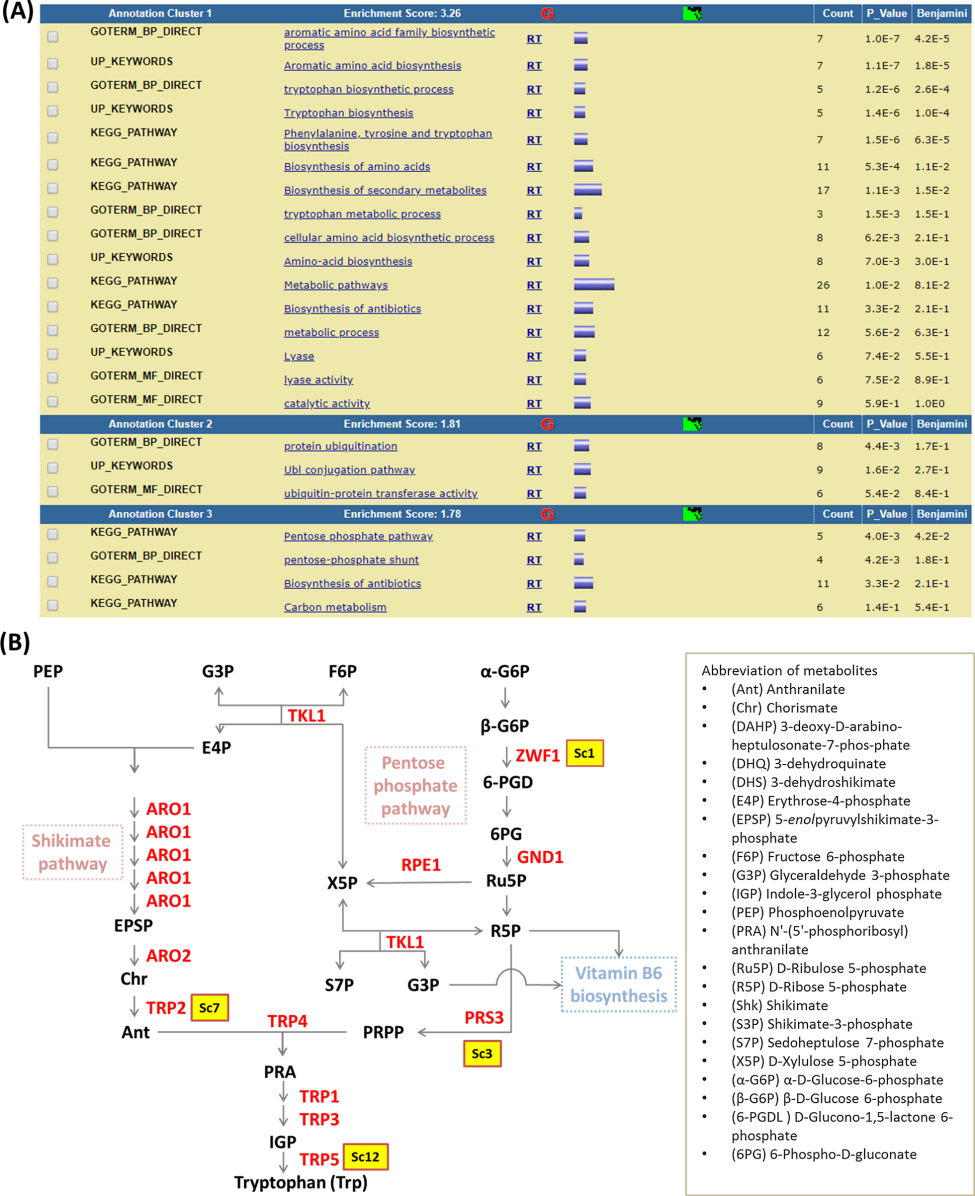
*S. cerevisiae* gene knock-out library screening for genes and pathways related to isobutanol tolerance. **a** Enrichment analysis was done by the online tool DAVID and the top 3 enriched pathways are listed here, including the tryptophan biosynthesis pathway (Annotation cluster 1), the ubiquitination pathway (Annotation cluster 2), and the pentose phosphate pathway (Annotation cluster 3). **b** Enriched biological functions including tryptophan biosynthesis and the pentose phosphate pathway. The genes related to isobutanol tolerance are labeled in red color

In order to identify genes related to isobutanol tolerance, an *S. cerevisiae* gene-deletion library (YKO MATa Strain Collection-Glycerol Stocks, Catalog #: YSC1053) was used for high-throughput screening. Each mutant strain was inoculated on solid medium plates with and without isobutanol treatment. The colony size was quantified after the plate image was scanned and recognized by the software “ScreenMill” [11]. Statistical analysis of colony images identified a series of mutants sensitive to isobutanol. Among the 5006 mutant strains assayed, a total of 161 gene-deletion clones showed a significant growth-reduction (Additional file 1: Table S1). An enrichment analysis (DAVID Bioinformatics Resources 6.8) led to cell functions enriched among the 161 genes, where the top 3 categories were genes engaged in tryptophan biosynthesis, ubiquitination, and the pentose phosphate pathway (Fig. 1a). The involvement of ubiquitination in *n*-butanol tolerance has been reported by González-Ramos et al*.* [12]. Their evolved *n*-butanol-tolerant strains also showed isobutanol tolerance, implying that *n*-butanol and isobutanol may induce similar responses from yeast cells. The ubiquitination gene cluster of our enrichment analysis included DNA damage-responsive RNA polymerase-degradation factor *DEF1*, E2 ubiquitin-conjugating protein *UBC8*, SCF ubiquitin ligase complex subunit *GRR1*, SUMO-targeted ubiquitin ligase complex subunit *SLX5*, SUMO-targeted ubiquitin ligase complex subunit *SLX8*, anaphase promoting complex subunit *DOC1*, ubiquitin-specific protease *UBP13*, and ubiquitin-specific protease *UBP15*. The genes *UCC1* and *UMP1* involved in the ubiquitin–proteasome system were also identified by González-Ramos et al*.* [12]. Our data suggest that isobutanol and *n*-butanol share similar tolerance mechanisms involved in protein degradation.

Our enrichment analysis also revealed a key cluster of defective genes in the tryptophan biosynthesis pathway. Pentafunctional AROM protein (*ARO1*), chorismate synthase (*ARO2*), anthranilate synthase (*TRP2*), anthranilate phosphoribosyltransferase (*TRP4*), phosphoribosylanthranilate isomerase (*TRP1*), indole-3-glycerol-phosphate synthase (*TRP3*), and tryptophan synthase (*TRP5*) were identified. In addition, genes clustered in the pentose phosphate pathway (PPP) also contributed to isobutanol tolerance. Those genes included glucose-6-phosphate dehydrogenase (*ZWF1*), phosphogluconate dehydrogenase (*GND1*), ribose phosphate diphosphokinase subunit (*PRS3*), ribulose-phosphate 3-epimerase (*RPE1*), and transketolase (*TKL1*) were identified. Interestingly, PPP is the main pathway to synthesize phosphoribosyl pyrophosphate (PRPP) and erythrose 4-phosphate (E4P), which are two important precursors for the shikimate pathway and tryptophan synthesis (Fig. 1b). These observations indicate that tryptophan or related metabolites may significantly contribute to isobutanol tolerance in yeast. It has been shown that tryptophan biosynthesis also responds to ethanol stress, while either overexpression or supplement of tryptophan could increase ethanol tolerance. Cellular membrane is a predominant target of ethanol, which decreases membrane integrity [13, 14]. Isobutanol is more hydrophobic than ethanol and is more toxic to cells by disrupting cell membrane. Stabilization of membrane has been found to protect yeast from isobutanol [15]. Tryptophan has been suggested to play a role in membrane stress response and to protect membrane from a common detergent sodium dodecyl sulfate (SDS) [16].

### Recovering and improving isobutanol tolerance by exogenous tryptophan

**Fig. 2:**
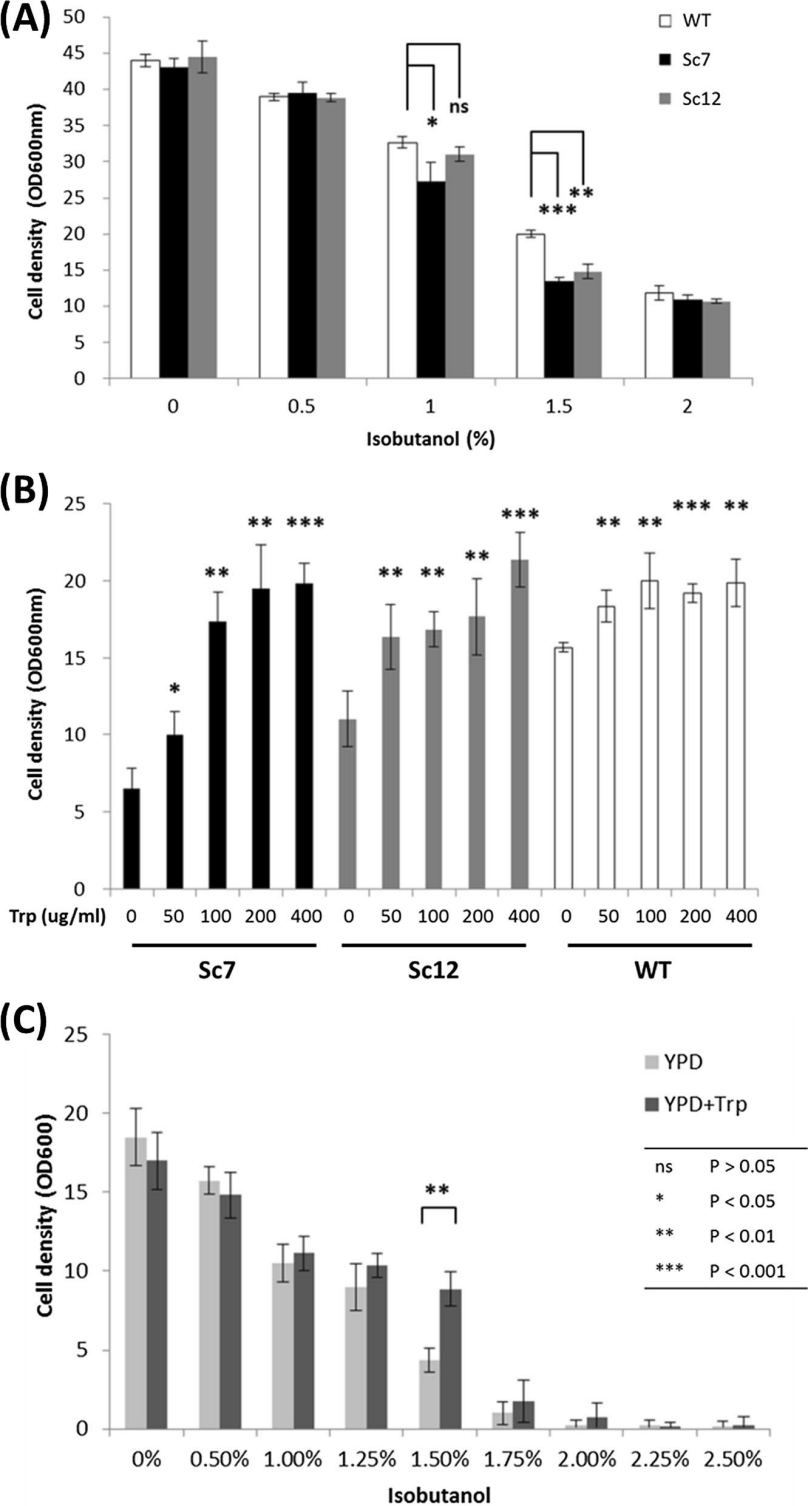
Growth assay related to the tryptophan biosynthesis pathway and isobutanol tolerance. **a** Growth assay (24 h) of the tryptophan biosynthesis pathway mutant strains Sc7and Sc12, and wild type (WT) in different isobutanol concentrations. Sc7 and Sc12 were more susceptible to isobutanol compared to WT. **b** The growth rate of defective mutant strains Sc7 and Sc12 can be recovered by adding exogenous tryptophan (24 h). **c** Wild type showed improvement in isobutanol tolerance after external tryptophan (200 μg/ml) was supplemented. The data represent the mean  ±  SD (*n*  =  3) (48 h)

To confirm the importance of tryptophan in isobutanol tolerance, the mutant strain Sc7 (Δ*TRP2*, anthranilate synthase deletion) and Sc12 (Δ*TRP5*, tryptophan synthase deletion) were used to conduct functional tests. TRP2 is a pivotal enzyme converting chorismate to anthranilate for the tryptophan biosynthesis pathway, and TRP5 is the final step to the tryptophan biosynthesis pathway. Growth assay was applied to different isobutanol concentrations. At the concentration of 1% and 1.5%, the mutant strains had a lower cell density than the wild type (Fig. 2a). This indicates that the defect of tryptophan mutants caused cells sensitive to isobutanol. A tryptophan supplementation assay was then introduced to examine whether the defect can be recovered or not. The result showed that mutant strains could be recovered by adding tryptophan in a dose-dependent manner (Fig. 2b). This also suggested that cells may become more sensitive to isobutanol when the concentration of tryptophan becomes low. Although the assay used normal medium to keep basic needs for cell’s living, the amount of tryptophan in medium is not enough for cells to tolerate isobutanol. We found that at least 100 µg/ml exogenous tryptophan was needed to recover the growth rate of the defective strain at its wild-type level. Thus, 200 µg/ml was applied in subsequent experiments. In addition, the wild type showed a slightly increased tolerance with tryptophan added in the medium. Therefore, the wild type was tested again to see whether tryptophan could improve the tolerance or not. Different concentrations of isobutanol treatment on the wild type were supplemented with and without tryptophan. In 1.5% isobutanol, the wild type fed with tryptophan had a higher survival rate compared to the sample without tryptophan supplement (Fig. 2c). Adding tryptophan to medium has not provided much improvement in the wild type, which may be because it still had a functional tryptophan pathway. In order to eliminate the nutritional growth effect from tryptophan metabolism, all strains were examined in conditions either with or without tryptophan addition. The result showed almost the same growth rate (Additional file 1: Figure S1). The above results indicated that tryptophan contributed to the increased tolerance of yeast to isobutanol.

### Tryptophan could recover isobutanol tolerance of defective PPP

**Fig. 3:**
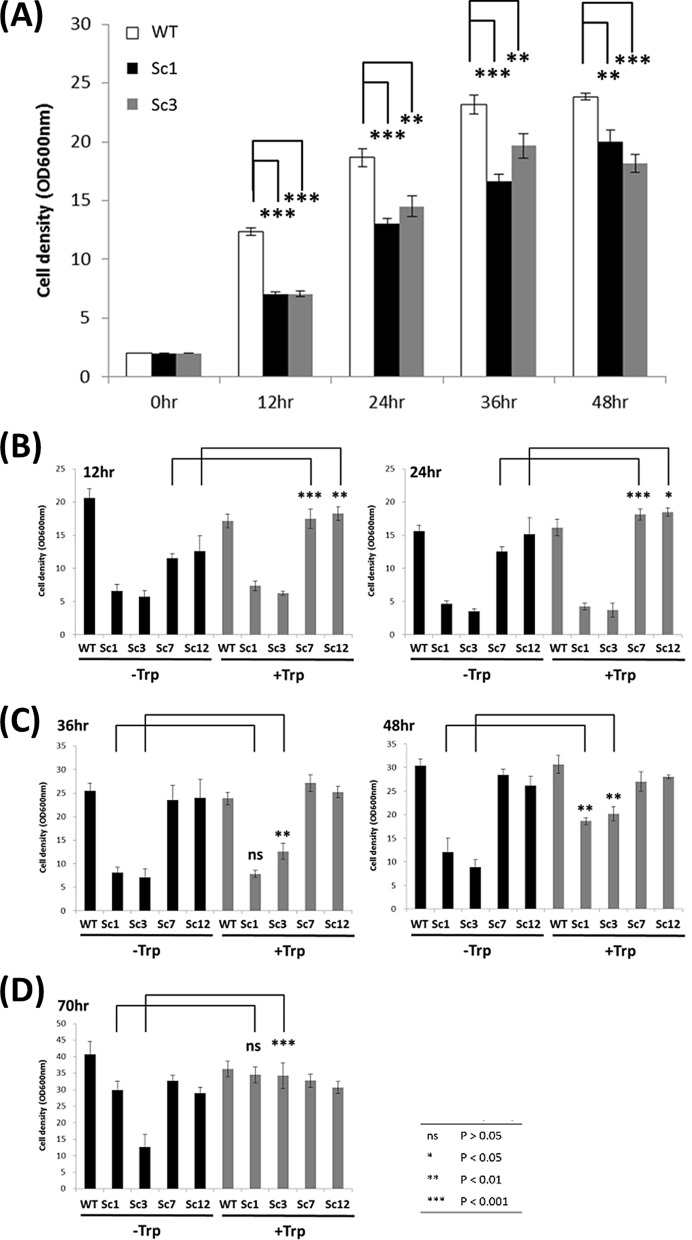
Growth assay related to the pentose phosphate pathway and isobutanol tolerance. **a** Growth assay was applied to two pentose phosphate pathway (PPP) defective strains (Sc1 and Sc3). The two mutant strains showed more sensitive to isobutanol (1%) compared to the wild type. **b**, **c** Two PPP-defective strains (Sc1 and Sc3) and two tryptophan pathway defective strains (Sc7 and Sc12) were used in recovery assays and the two tryptophan pathway defective strains were recovered earlier **(b)** than the two PPP-defective strains **(c)**. **d** All defective strains were recovered at 70 h. The recovery assays was performed at 200 μg/ml tryptophan addition

The pentose phosphate pathway (PPP) is an essential pathway to provide tryptophan precursors E4P and PRPP. In initial tryptophan biosynthesis, phosphoenolpyruvate (PEP) derived from glycolysis is condensed with E4P and enters into the shikimate pathway to form chorismate. Chorismate is then converted into anthranilate, which then condenses with PRPP for the tryptophan biosynthesis pathway. PPP plays a crucial role in tryptophan biosynthesis and we found that a defect in PPP makes yeast sensitive to isobutanol. The mutant strains Sc1 (Δ*ZWF1*, glucose-6-phosphate 1-dehydrogenase deletion) and Sc3 (Δ*PRS3*, ribose-phosphate pyrophosphokinase deletion) were selected for further characterization. ZWF1 is the first rate-limiting step to introduce the metabolic flux into PPP, and PRS3 is a key enzyme to produce PRPP for tryptophan biosynthesis. Sc1 and Sc3 grew more slowly under isobutanol treatment than the wild type, and both could be recovered by adding tryptophan (Fig. 3a; Additional file 1: Figure S2). However, when we checked the recovery time, Sc1/Sc3 recovered more slowly than Sc7/Sc12 (Fig. 3b, c). This suggests that tryptophan can rescue tryptophan biosynthesis defect more directly than PPP defect. Thus, the PPP pathway seems to be involved in isobutanol tolerance through the tryptophan pathway. Sc3 showed most severe growth defect compared to the other 3 mutant strains, but it could be recovered by adding tryptophan. The result suggests that PRPP for tryptophan biosynthesis is important for isobutanol tolerance (Fig. 3d). For this reason, we subsequently focused on profiling the genetic responses, and surveyed the relationships between isobutanol stress and tryptophan through transcriptomic analysis by next-generation sequencing.

### Transcriptional responses to isobutanol stress and exogenous tryptophan

**Fig. 4:**
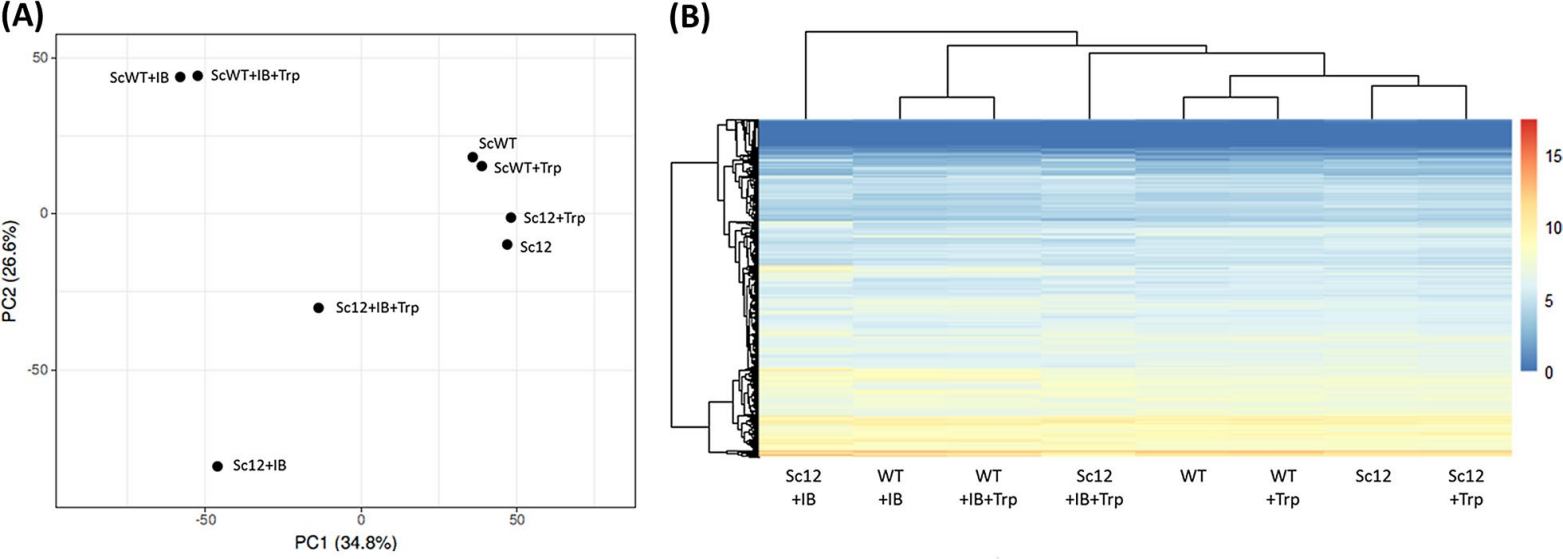
Transcriptomic analysis of the tryptophan pathway under isobutanol stress. The wild type and tryptophan-defective strains were studied under four different conditions: with/without isobutanol and/or supplemental tryptophan. Total RNAs were harvested after 24 h for RNA sequencing. **a** Principal component analysis (PCA) using the RPKM data. Each spot represents 6933 genes’ expression levels. In terms of the distance between spots, the wild type and the mutant strain were not very different under no isobutanol stress, but had large differences under isobutanol stress. Adding external tryptophan mitigated the effect of isobutanol stress on the mutant strain. **b** Heatmap and hierarchical clustering showed similar result as the PCA plot

To evaluate the cellular response to isobutanol and tryptophan, transcriptomic analysis was conducted to characterize the transcriptome profile and pathway regulation. The wild type and mutant strain Sc12 were subject to 4 different conditions for 24 h: no treatment, tryptophan treatment, isobutanol treatment, and isobutanol plus tryptophan. Each sample was obtained from three replicates, growth conditions were checked before RNA harvesting (Additional file 1: Figure S3), and knockout of *TRP5* was verified by polymerase chain reaction (PCR) and RNA sequencing (Additional file 1: Figure S4a, b). The sequencing reads were mapped to the reference genome (UCSC sacCer3) by Tophat (Additional file 1: Table S2), and the FPKM (fragments per kilobase of transcript per million) was calculated by Cufflinks. To characterize gene expression patterns, heatmaps and principal component analysis (PCA) plots were obtained in terms of FPKM values (Fig. 4a, b). Based on the PCA plots and the heatmaps, the Sc12 strain treated with isobutanol was very different from the other samples. When external tryptophan was added to Sc12 with isobutanol, the gene expression pattern shifted closer to the normal condition, suggesting that tryptophan mitigated the effects of isobutanol (Sc12  +  IB vs. Sc12  +  IB  +  Trp). On the other hand, the wild type showed only a slight change in expression pattern when tryptophan was added to the medium that contained isobutanol (WT  +  IB vs. WT  +  IB  +  Trp). Furthermore, both the wild type and the mutant strain did not show much change in the transcriptome when supplemented with tryptophan (WT vs. WT  +  Trp/Sc12 vs. Sc12  +  Trp). The result suggests that tryptophan did not significantly alter the growth of WT and Sc12, but specifically mitigated the effect of isobutanol on Sc12. The overall transcriptome pattern seems to reflect the growth assay, and was reliable for further differential expression analysis. Table 1 shows the number of differentially expressed genes (DEGs) for each pair and the cutoff threshold was set to *q* value  =  0.05 and fold-change  =   ±  2. The DEG numbers of WT versus WT  +  Trp (7) and Sc12 versus Sc12  +  Trp (145) were small, implying that exogenous tryptophan did not have much effect on growth. In addition, when we compared WT  +  IB and WT  +  IB  +  Trp, only 14 genes showed significant changes in expression level, suggesting that external tryptophan has no significant effect on the gene expression of the wild type. In order to know the biological functions in which the DEGs participated, enrichment analysis of all paired groups was done. The enriched pathways mainly include biosynthesis of amino acids, the pentose phosphate pathway, tryptophan metabolism, the nicotinamide adenine dinucleotide (NAD) biosynthetic process, fatty acid metabolism, thiamine (Vitamin B_1_) metabolism, and vitamin B_6_ metabolism. The gene expression level of each enriched pathway was subject to cluster analysis to reveal their relationships.

**Table 1:** Number of differentially expressed genes (DEGs) between each sample pair

	WT	WT + Trp	WT + IB	WT + IB + Trp	Sc12	Sc12 + Trp	Sc12 + IB	Sc12 + IB + Trp
WT	–				408			
WT + Trp	7	–				290		
WT + IB	1374		–				1628	
WT + IB + Trp		1313	14	–				1379
Sc12					–			
Sc12 + Trp					145	–		
Sc12 + IB					1768		–	
Sc12 + IB + Trp						1361	1506	–

The cutoff threshold was set up as *q* value  =  0.05 and fold-change  =   ±  2

*Trp* tryptophan; *IB* isobutanol

#### Biosynthesis and transportation of amino acids responding to isobutanol stress in a strain with a defective tryptophan biosynthesis pathway

**Fig. 5:**
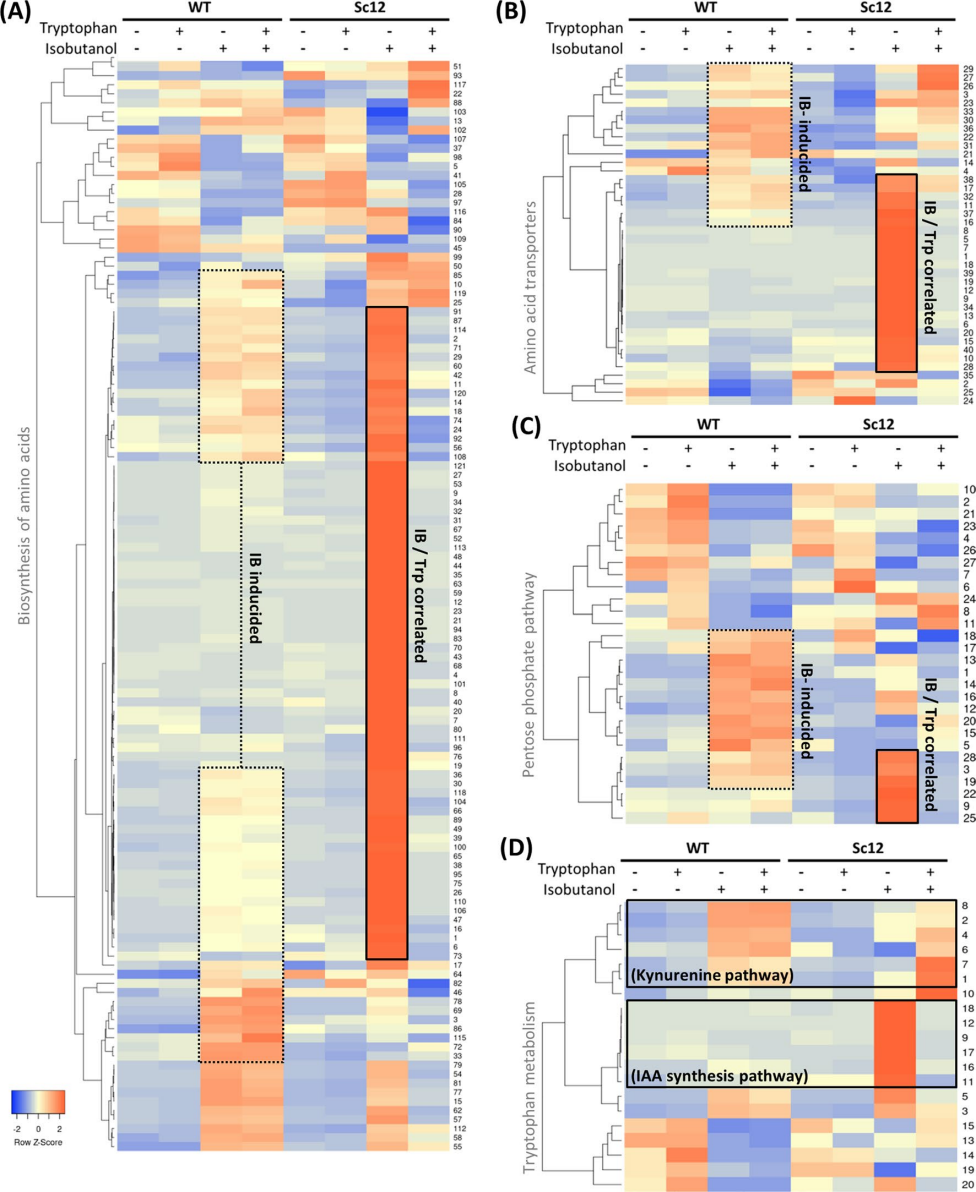

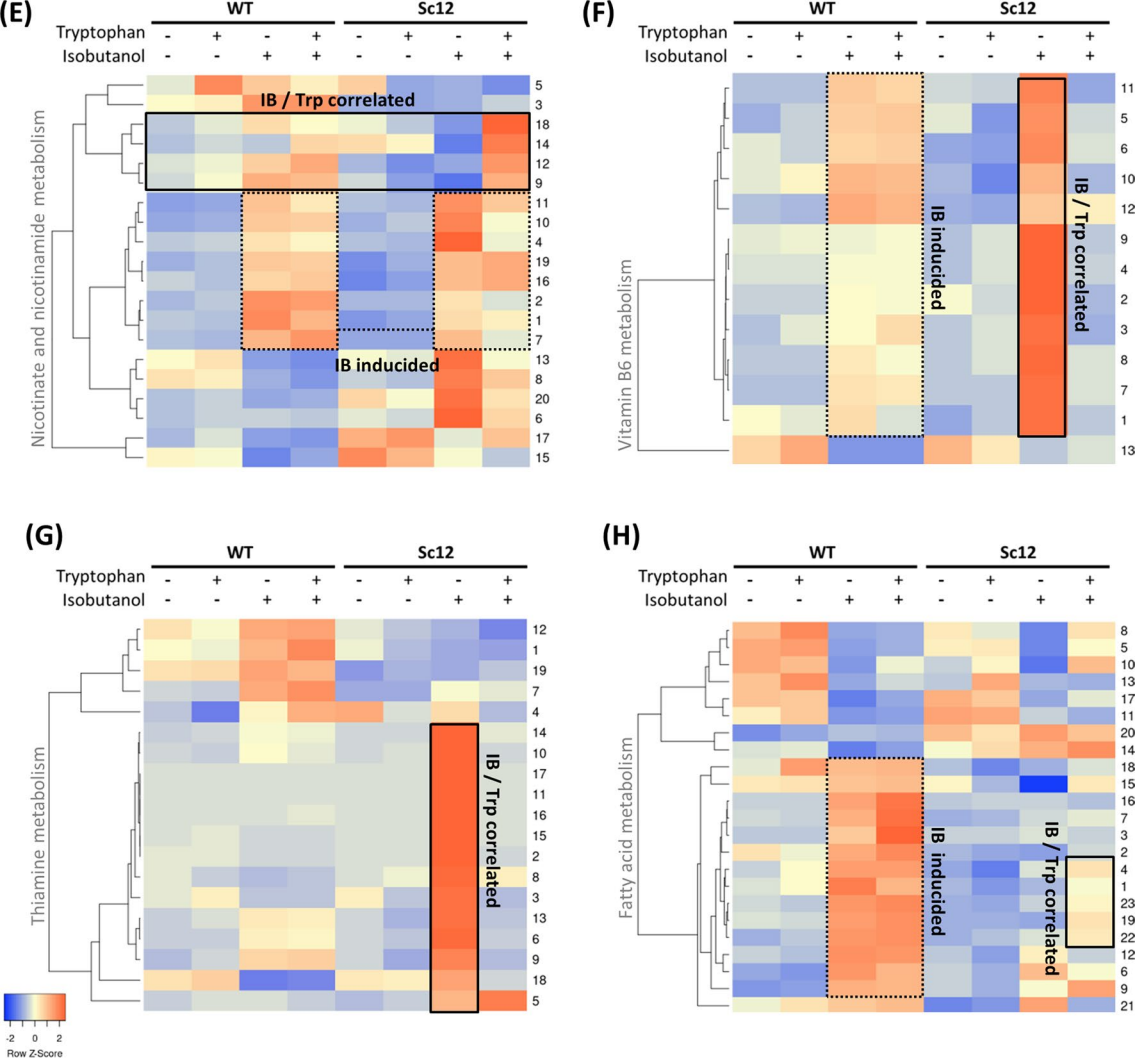
Clustering analysis of enriched cell functions related to isobutanol stress and tryptophan supplementation. The expression changes between each sample pair were subject to enrichment analysis. The enriched cellular functions were then clustered to exhibit the gene expression pattern differences between the high and low expression levels in each sample, which were represented by red and blue color, respectively. The enriched cell functions include: **a** biosynthesis of amino acids, **b** amino acid transporters, **c** the pentose phosphate pathway, **d** tryptophan metabolism, **e** nicotinate and nicotinamide metabolism, **f** vitamin B_6_ metabolism, **g** thiamine (vitamin B_1_) metabolism, and **h** fatty acid metabolism. The rectangle emphasizes the gene clusters related to isobutanol stress and tryptophan. The number to the right-hand side represents every single gene applied to each heatmap. Gene names of each heatmap are listed in Additional file 1: Table S3

Biosynthesis of amino acids seems to play an important role in mitigating the effect of isobutanol stress when the tryptophan biosynthesis pathway is defective. The cluster analysis showed that many genes participating in amino acid biosynthesis were highly expressed when wild type was treated with isobutanol. Compared to wild type, the mutant strain Sc12 showed stronger expression pattern when treated with isobutanol, but were then down-regulated by tryptophan supplementation (Fig. 5a). The up-regulated biosynthesis pathways included Ala, Gly, Ser, Pro, His, Phe, Tyr, Trp, Val, Leu, Ile, Cys, Met, Thr, Asp, Asn, Lys, Glu, Gln, and Arg, suggesting that cells require a broad range of amino acids to tolerate isobutanol stress when the tryptophan biosynthesis pathway is defective. In addition, about half of amino acid transporters showed high expression under isobutanol stress. Other transporters were up-regulated in Sc12 under isobutanol stress and down-regulated after adding tryptophan, in which almost all-natural amino acid transporters directed amino acids from extracellular to cytosol (Fig. 5b). These observations indicate that the cells try not only to synthesize but also to import amino acids, and tryptophan seems to play a key role in this process. Meanwhile, transketolase *TKL1* (YPR074C) and transaldolase *TAL1* (YLR354C) were slightly over-expressed to activate the pentose phosphate pathway (PPP) non-oxidative phase, trying to awake tryptophan biosynthesis upstream in the defective strain, and were down-regulated after adding tryptophan. In addition, some PPP-related genes were highly expressed under isobutanol stress in the wild type, but down-regulated in the mutant strain (Fig. 5c). Those genes included Sol2p (*SOL2*), 6-phosphogluconolactonase 3 (*SOL3*), phosphogluconate dehydrogenase (*GND1*), ribulose-phosphate 3-epimerase (*PRE1*), ribose-phosphate pyrophosphokinase 1 (*PRS1*), ribose-phosphate pyrophosphokinase 3 (*PRS3*), ribose-phosphate pyrophosphokinase 4 (*PRS4*), and phosphoglucomutase (*PGM1*). The high expression of *TKL1*, *GND1*, *RPE1*, and *PRS3* under isobutnaol stress confirmed the result in knock-out strains screening, in which deletion of any of these genes was susceptible to isobutnal. This supports the view that the PPP is related to the tryptophan pathway for isobutanol tolerance.

#### Tryptophan downstream metabolism also plays an important role in yeast’s tolerance to isobutanol

Tryptophan metabolism involves processes to convert tryptophan to many downstream bioactive molecules such as melatonin, kynurenine, indole-3-acetic acid (IAA), and nicotinamide adenine dinucleotide (NAD^+^). Cluster analysis of genes involved in tryptophan metabolism resulted in two groups, one enriched in the kynurenine pathway and the other enriched in the IAA biosynthesis pathway (Fig. 5d). Genes in the kynurenine pathway, such as tryptophan 2,3-dioxygenase (*BNA2*), formylkynurenine formamidase (*BNA7*), and kynureninase (*BNA5*), were highly expressed when WT was treated with isobutanol, but were down-regulated when the mutant strain Sc12 was treated with isobutanol. After adding tryptophan to Sc12, genes in the kynurenine pathway were up-regulated, implying that the activation of the kynurenine pathway requires tryptophan. The kynurenine pathway of tryptophan metabolism exhibits antioxidant property to eliminate oxidative stress, especially using the enzyme indoleamine 2,3-dioxygenase (IDO) [17, 18]. IDO, named BNA2 in *S. cerevesia*, was also highly expressed when WT was treated with isobutanol but down-regulated in Sc12 when it was treated with isobutanol, and it could be up-regulated by adding tryptophan (Additional file 1: Figure S5). In addition, the kynurenine pathway is the only de novo biosynthesis pathway of nicotinamide adenine dinucleotide (NAD). NAD^+^ can be phosphorylated to NADP^+^ by NAD^+^ kinase, which can be reduced to NADPH and NADH, respectively. NAD(H) is important in regulating cellular energy metabolism, and NADP(H) is mainly involved in redox balance and biosynthesis of fatty acids and nucleic acids [19]. In the cluster analysis of nicotinate and nicotinamide metabolism, a large proportion of genes were up-regulated when the cell was treated with isobutanol. In Sc12, some of the genes did not respond to isobutanol and were reactivated when adding tryptophan. These genes, including nicotinic acid mononucleotide adenylyltransferase (*NMA2*) and glutamine-dependent NAD synthetase (*QNS1*), could downregulate NAD^+^ production when Sc12 was under isobutanol stress (Fig. 5e), and the expression pattern was similar to the genes in the kynurenine pathway, implying that cells rely on tryptophan to synthesize NAD^+^ through the kynurenine pathway. Thus, the function of NAD^+^, such as redox balance regulation for fatty acid synthesis and anti-oxidative ability, may contribute to isobutanol tolerance.

In the IAA biosynthesis pathway, genes were up-regulated only when the mutant strain was under isobutanol stress, and then returned to low-level expression after tryptophan addition (Fig. 5d). This is similar to the expression pattern of amino acid biosynthesis and transport. IAA is a common natural compound in plants and microbes, and regulates plant-microbe interaction. IAA can stimulate plant cell elongation, division, and differentiation. Moreover, exogenous IAA can regulate adhesion and filamentation in *S. cerevisiae* by inducing morphological genes [20, 21]. This is consistent with the finding that filamentous growth could increase yeast isobutanol tolerance [22]. However, we did not observe obvious morphological change in both WT and Sc12 when grown under isobutanol. Although up-regulation of the IAA pathway seems to convert upstream tryptophan into downstream IAA, the occurrence of this phenomenon in the defective tryptophan biosynthesis pathway strain makes it difficult to explain. Although there is evidence that IAA has a tryptophan-independent biosynthesis pathway, which branches from either indole or indole-3-glycerol phosphate, the specific enzymes in *S. cerevisiae* still have not been identified [23]. The actual role of IAA and its regulation of biosynthesis pathway under isobutanol stress remain to be investigated.

#### Vitamins B_**6**_ and B_1_ support isobutanol tolerance

Induction of vitamin B_6_ and vitamin B_1_ (thiamine) metabolic genes were observed under isobutanol stress. Vitamin B_6_ biosynthesis genes were up-regulated when the WT was treated with isobutanol. Moreover, similar to amino acid biosynthesis and tryptophan metabolism, both vitamin B_6_ and B_1_ genes showed a high expression level when the mutant strain Sc12 was under isobutanol stress, indicating that vitamin B_6_ and B_1_ genes respond to isobutanol stress and are related to tryptophan (Fig. 5f, g). Vitamin B_6_ is a group of similar biological active compounds including pyridoxine (PN), pyridoxal (PL), and pyridoxamine (PM), and their phosphorylated derivatives pyridoxine 5′-phosphate (PNP), pyridoxamine 5′-phosphate (PMP), and pyridoxal 5′-phosphate (PLP). PLP is the active form of vitamin B_6_ and plays as a coenzyme or substrate for many bioprocesses such as amino acid biosynthesis, lipid metabolism, and thiamine biosynthesis. The precursors of PLP are converted from the PPP intermediates (glyceraldehyde 3-phosphate and ribose 5-phosphate) and glutamine. Their related enzymes, *TKL1*, *TAL1*, and *GLN1* (glutamine synthetase), exhibited similar gene expression patterns to vitamin B_6_ and might facilitate the biosynthesis of vitamin B_6_. PLP is also needed for many aminotransferases and synthases, such as tryptophan synthase (*TRP5*), and this observation may explain the requirement of amino acids when a mutant strain is under isobutanol stress. Furthermore, kynureninase (*BNA5*) is a PLP-dependent enzyme to facilitate downstream tryptophan metabolism such as NAD^+^ de novo synthesis. PLP can be further combined with histidine and thiazole moiety, derived from glycine and NAD^+^, to form vitamin B_1_. The major active derivative of vitamin B_1_ is thiamine diphosphate (TDP) and serves as a cofactor for many bioprocesses such as TKL1 in PPP [24]. However, when exogenous vitamin B1 (thiamine) was added to medium in tolerance assay, there was no improvement in cell density under isobutanol stress (Additional file 1: Figure S6). This may be because, as a cofactor, vitamin B1 may not work alone to rescue the tryptophan-defective strain (Sc12). This issue requires further study.

To summarize, the enriched pathways described above seem to coordinate with each other when a cell is under isobutanol stress. Yeast tries to optimize the expression levels of genes participating in the biosynthesis or import of amino acids under conditions such as nitrogen starvation. The tryptophan metabolism pathway is activated and induces IAA biosynthesis, the kynurenine pathway, and nicotinamide metabolism. Nicotinamide metabolism may provide redox pool (NAD^+^/NADP^+^) and the pentose phosphate pathway (PPP) may support anti-oxidative ability. The kynurenine pathway also plays an important role against free oxidative stress. PPP, NAD^+^, histidine and glycine provide raw materials for the synthesis of vitamins B_6_ and B_1_, which can then enhance amino acid biosynthesis, tryptophan metabolism, and PPP. Fatty acid metabolism genes are also up-regulated to confront isobutanol in the WT, and those genes mainly participate in fatty acid biosynthesis. This implies that fatty acid biosynthesis may help tolerate solvent stress, such as strengthening the cell membrane. In addition, some genes were down-regulated in the mutant strain and could be rescued by tryptophan, especially acetyl-coA carboxylase (*ACC1*), which is a rate-limiting step for the biosynthesis of fatty acids (Fig. 5h). The expression patterns of fatty acid synthesis genes are similar to those in the kynurenine pathway and NAD^+^ de novo synthesis. Accordingly, tryptophan and tryptophan metabolism seems to play a role in coordinating fatty acid biosynthesis. However, the relationship between tryptophan and fatty acid synthesis is not yet clear in yeast. The increased NAD pool from tryptophan metabolism may be a reason that it provides energy for fatty acid biosynthesis for emergency. Based on our transcriptomic analysis, many biological networks seem to work together to build a defense wall to protect cells from isobutanol stress.

## Discussion

A high-throughput platform was built in this study to identify isobutanol-susceptible mutants through their colony size. A total of 5006 single-gene deletion strains were tested and 161 gene-deletions exhibited reduced growth. To understand what biological functions may be involved in isobutanol tolerance, functional enrichment analysis was applied to these 161 genes. The result indicated that tryptophan biosynthesis, ubiquitination, and PPP were the top three enriched functions. By adding exogenous tryptophan to a tryptophan biosynthesis or PPP-defective strain, the isobutanol tolerance can be recovered, implying that tryptophan is a key element in yeast’s isobutanol tolerance. Kuroda et al. [10] have previously shown that tryptophan and PPP were involved in isobutanol tolerance. Their study revealed the key role of tryptophan in PPP/GLN3 and nitrogen starvation response while we provided insights into the tryptophan pathway. Furthermore, we revealed the potential relationship between tryptophan and nitrogen starvation response. Our results support the finding of Kuroda et al. [10], and suggest the possible network behind the “isobutanol stress-induced” nitrogen starvation.

In order to understand the genetic basis of cellular functions participating in the stress tolerance, transcriptome analysis was performed to distinguish expression profiles between samples treated with/without isobutanol and/or tryptophan. Principal component analysis (PCA) and hierarchical clustering were employed to show that the defective tryptophan biosynthesis strain exhibited large differences in transcriptome patterns from the wild type, and the differences were reduced by exogenous tryptophan. Enrichment analysis and cluster analysis revealed that amino acid biosynthesis and transportation were highly expressed under isobutanol stress. The results showed a higher level of amino acid biosynthesis in the tryptophan biosynthesis-defective strain than the wild type. In contrast, the high expression level was reduced by adding tryptophan. These observations suggest that the activation of amino acid biosynthesis is closely related to the defect in tryptophan biosynthesis. In addition, a large number of amino acid transporters were also up-regulated by isobutanol when tryptophan biosynthesis was defective. These results point to the emergency demand of amino acids, similar to the hypothesis of nitrogen starvation response induced by isobutanol proposed by Kuroda et al*.* [10]. Their transcriptomic analyses revealed that isobutanol induces a nitrogen starvation response via *GLN3* [10]. In our transcriptomic data, the tryptophan biosynthesis mutant and its isobutanol-treated sample had increased *GLN3* expression that could be reduced by adding external tryptophan (Additional file 1: Figure S7). This implies that the *GLN3* expression level is affected by tryptophan under isobutanol stress. Furthermore, it has been shown that the level of glutamine is a key indicator of nitrogen starvation, which can activate the transcription factor *GLN3* [25]. In both *S. cerevisiae* and *E. coli*, glutamine level drastically dropped under nitrogen starvation. Under such condition, synthesis of the aromatic amino acids including phenylalanine, tyrosine, and tryptophan can follow a common pathway upstream to the key intermediate chorismate, which contains no nitrogen. Chorismate could then acquire nitrogen from glutamine to continue the tryptophan pathway [26]. In our transcriptome data, the expression levels of glutamine synthetase (*GLN1*) and vacuolar glutamine transporter (*Avt7*) were up-regulated under isobutanol stress to improve glutamine synthesize and homeostasis. The glutamine transporters, glutamine permease (*GNP1*) and dicarboxylic amino acid permease (*DIP5*), located on plasma membrane were all up-regulated when tryptophan biosynthesis-defective strains confront isobutanol stress, which may support the demand of glutamine (Additional file 1: Figure S8). These lines of evidence suggest an important role of glutamine in “isobutanol stress-induced” nitrogen starvation, in particular when cells consume more tryptophan under isobutanol stress. Furthermore, in Kuroda et al. [10], the *gln3*Δ strain showed lower intracellular glutamine concentration compared to the wild type regardless of the presence or absence of isobutanol. This suggests that downstream genes of *GLN3* may upregulate the ratio and concentration of glutamine/glutamate. When under isobutanol stress, *GLN3* may try to satisfy the emergent demand of glutamine.

Hence, we postulate that the tryptophan demand increased in response to isobutanol stress, triggering the need for an additional nitrogen donor, in particular glutamine. Meanwhile, the decreasing glutamine level may activate the transcriptional regulator *GLN3* for further nitrogen starvation response. The difference between “true” nitrogen starvation and “isobutanol stress-induced” nitrogen starvation is that under true nitrogen starvation there is no nitrogen supply from glutamine whereas in the “isobutanol stress-induced” nitrogen starvation cells can take advantage of chorismate and acquire nitrogen from glutamine. The expression profile of *GLN3* in Additional file 1: Figure S7 supports our hypothesis and largely explains why the expression level of *GLN3* did not change when the wild type was treated with isobutanol. In addition, when a cell has a defective tryptophan pathway, *GLN3* and the glutamine-related genes (*GLN1*, *DIP5*, and *GNP1*) were highly up-regulated (Additional file 1: Figures S7, 8) and the growth defect became more severe than the wild type (Additional file 1: Figure S3). After the addition of exogenous tryptophan, the expression levels of *GNL3* and the glutamine-related genes were decreased and the growth defect was recovered (Additional file 1: Figure S3). These results were consistent with the previous finding that the *GLN3* knock-out strain showed better tolerance to isobutanol [10].

On the other hand, ubiquitination was enriched in our knockout screening analysis, which is consistent with the result of González-Ramos et al. [12], indicating that protein degradation and turnover have been activated during the butanol challenge in *S. cerevisiae*. The biologically active B_6_ vitamin PLP is the coenzyme involved in protein synthesis, degradation, and amino acid interconversion. Indeed, the gene expression patterns of amino acid synthesis and their transporters (Fig. 5a, b) and vitamin B_6_ synthesis (Fig. 5f) appeared highly allied. Thus, we suggest that protein turnover may facilitate isobutanol tolerance in yeast by providing free amino acids as a response to nitrogen starvation.

It is interesting that the exogenous tryptophan rescued the PPP defect more slowly than the tryptophan pathway defect in yeast. Many biochemical reactions rely on PPP as a precursor supplier. PPP is not only an important upstream pathway of tryptophan biosynthesis, but also is needed for the cofactors NAD/NADP, nucleotides, and some other amino acids biosynthesis. In addition, PPP also provides glyceraldehyde 3-phosphate and R5P as precursors for vitamin B_6_ synthesis, while vitamin B_6_ is involved in the reactions of tryptophan synthase (*TRP5*) and kynureninase (*BNA5*). Thus, PPP plays multiple roles in both upstream tryptophan biosynthesis and downstream tryptophan metabolism. We speculate that although tryptophan supplementation does not immediately rescue the defect in PPP, exogenous tryptophan may reduce the consumption of PPP intermediates used for isobutanol tolerance. The conservation of more PPP intermediates can be used for other biosynthesis that is needed for cell survival. As a result, cell growth of the PPP-defective strain is gradually recovered by tryptophan supplementation. This could explain why the tryptophan rescue on PPP mutants occurred much later than that in tryptophan mutants. Our view is supported by Kuroda et al. [10] report that deletion of PPP genes *GND1* or *ZWF1* causes hypersensitivity to isobutanol. In addition, PPP gene expression pattern was similar to both tryptophan and vitamin B_6_/B_1_ biosynthesis, suggesting that PPP might support the synthesis of precursors for tryptophan and vitamin B_6_/B_1_ under isobutanol stress. Furthermore, TPP-dependent enzyme transketolase *TKL1* (YPR074C) was over-expressed in isobutanol-treated Sc12, which is also consistent with the finding of vitamin B_1_ activation. Oxidative and osmotic stresses increased the production of numerous vitamin B_1_ biosynthesis enzymes in yeast, indicating a protective role of vitamin B_1_ against adverse environmental conditions. Mutations of vitamin B_1_ synthesis genes increased the sensitivity to oxidative stress, suggesting that thiamine metabolism can partly compensate damages of yeast general defense systems [27].

The tryptophan mutant strain seemed to have reduced gene expression related to the kynurenine pathway. The kynurenine pathway in tryptophan metabolism is the only de novo biosynthesis pathway of nicotinamide adenine dinucleotide (NAD). This suggests that tryptophan may activate the kynurenine pathway and nicotinamide metabolism for isobutanol tolerance. Over-expressing the first biosynthesis of NAD  +  (kynurenine) pathway gene *BNA2* skews glycolytic flux from lipid droplets towards the aromatic amino acid biosynthesis-BNA pathways. This reduces lipid droplet accumulation during aging and extends lifespan of yeast [28]. Kynurenine exhibits antioxidant property that eliminates oxidative stress using the enzyme indoleamine 2,3-dioxygenase (IDO). IDO is a unique enzyme that utilizes superoxide anion radical (O_2_*-) as both a substrate and a cofactor. The antioxidant activity may arise from O_2_*-scavenging by IDO and formation of the potent radical scavengers and kynurenine pathway metabolites, 3-hydroxyanthranilic acid and 3-hydroxykynurenine [18, 29]. Therefore, IDO has been proposed to be an antioxidant enzyme [30]. In addition, PPP may also provide energy to NADPH for anti-oxidative ability. This hypothesis is supported by Kuroda et al. [10] observation that the NADPH/NADP  +  ratio was decreased in PPP-defective strains (*zwf1*Δ). Furthermore, the isobutanol-specific sensitivity of PPP non-oxidative phase deletion (*tkl1*Δ) observed by Kuroda et al. [10] can be explained by our hypothesis that the upstream genes of tryptophan biosynthesis in PPP contribute to isobutanol tolerance. This also suggests that both of PPP and tryptophan metabolism may attenuate oxidative stress created by isobutanol.

Furthermore, the generation of NAD(P) from the kynurenine pathway is not only important in redox balance but also involved in the biosynthesis of fatty acids [31] that are needed for cell membrane formation. Fatty acid is the major source for cell membrane, so it may contribute to isobutanol tolerance. Isobutanol induced many fatty acid synthesis genes in WT, though not in the Sc12, regardless of tryptophan supplementation (Fig. 5h). These results indicate that tryptophan synthase is essential for isobutanol-induced fatty acid synthesis. Despite that adding tryptophan did not recover all down-regulated genes in the mutant strain Sc12, some key genes were rescued by exogenous tryptophan, such as *ACC1*, which catalyzes the rate-limiting step of fatty acid biosynthesis (Additional file 1: Figure S9). Thus, we postulate that tryptophan enhances cell membrane integrity via fatty acid synthesis. Tryptophan supplementation to culture and/or overexpression of the tryptophan permease gene has been found to be effective for increasing ethanol stress tolerance [32]. Tryptophan confers the *S. cerevisiae* resistance to SDS-induced cell membrane disruption. Overexpression of tryptophan permease gene *TAT2* rescues cell growth in SDS-treated Δ*mck1* cells [16], and *TAT2* confers high-pressure growth in *S. cerevisiae* [33]. Tryptophan import was enhanced upon yeast exposed to SDS, and internal concentration of tryptophan makes cells more resistant to membrane disruption caused by sodium dodecyl sulfate [16]. Our data also showed the enhancement of *TAT2* expression under isobutanol stress (Additional file 1: Figure S10). In addition, although there is no morphological change in our observation, we found that the IAA biosynthesis pathway was increased in the tryptophan-defective strain under isobutanol stress, and IAA may be related to the filamentous phenotype. Although the literature rarely described the mechanism of tryptophan on yeast cell wall/membrane protection, the above studies implicate that tryptophan may contribute to cell wall/membrane integrity during isobutanol stress.

In summary, the defense of yeast against isobutanol stress is highly dependent on tryptophan and related networks. Under isobutanol stress, the depletion of tryptophan may acquire glutamine as the supplement of nitrogen followed by the chorismate pathway. Consequently, the lower level of glutamine alters *GLN3* expression and triggers the nitrogen starvation responses which enable the cells to manage the stress by activation of protein turnover, amino acid synthesis and import that helps replenish glutamine and tryptophan. Our transcriptomic analysis also revealed the correlated cell functions surrounding tryptophan, such as the kynureninase pathway of tryptophan metabolism and nicotinamide metabolism. These metabolisms may provide a NAD(P) pool, while PPP roles as the main resource to convert NADP to NADPH for anti-oxidative ability and fatty acid synthesis. Vitamins B_6_ and B_1_ synthesis genes may be up-regulated to assist these metabolic processes including amino acid biosynthesis, PPP, and lipid metabolism. Our results demonstrate the dynamics of yeast cell under isobutanol stress and tryptophan is important for its tolerance.

## Conclusions

A high-throughput colony screening method provided an efficient way to reveal the key biological functions involved in isobutanol tolerance. The growth of strains defective in either the tryptophan biosynthesis pathway or the PPP pathway could be recovered by exogenous tryptophan when they were under isobutanol stress, implying the importance of tryptophan biosynthesis in yeast’s isobutanol tolerance. Based on the cluster analysis of transcriptomic data, many cell functions including amino acid biosynthesis/transport, tryptophan metabolism, vitamin B_6_ and B_1_ biosynthesis, and fatty acid metabolism were found to coordinate with each other under isobutanol stress. Based on this and previous studies, we suggest that the *GLN3*-induced nitrogen starvation may be due to depletion of tryptophan and its nitrogen donor glutamine. Our study highlights the central role of tryptophan in yeast’s tolerance to isobutanol and offers new clues for engineering yeast with higher isobutanol tolerance.

## Methods

### Growth assay

*Saccharomyces cerevisiae* BY4741 was used in this study. All strains under study were grown in the YPD medium composed of yeast extract 10 g/L, peptone 20 g/L, and glucose 20 g/L. G418 (200 µg/mL) was added into YPD to maintain the Yeast Knockout Collection. Cells were thawed from a − 80℃ stock and were precultured in fresh medium for 24 h before assay. The isobutanol tolerance assay was conducted in the YPD medium with/without tryptophan (200 µg/ml). After 24 h, the cell density was measured by optical density at a wavelength of 600 nm using disposable cuvettes (10 mm). In recovery assay, cells were cultured in YPD medium with 1% isobutanol and different concentrations of tryptophan, and cell density was measured after 24 h. The wild-type tolerance assay was tested by adding 200 µg/ml tryptophan and cell density was measured after 24 h and 48 h. The recovery assay monitor at different time points was tested with/without 200 µg/ml tryptophan under 1% isobutanol stress. In colony assay, the yeast strains were inoculated in YPD liquid medium with 1% Isobutanol for 24 h, and then were serially diluted on YPD agar plates with 5 μl loading volume for 48 h incubation. The growth temperature was set at 30 ℃.

### Screening the yeast gene-deletion library

YKO MATa Strain Collection-Glycerol Stocks (Catalog #: YSC1053) was used as a gene-deletion library to screen isobutanol-sensitive mutant strains. The square plate composed of YPD agar with/without 2% isobutanol was prepared for colony array inoculation. The deletion library was precultured in 96-well plate with 200 µl medium (30 °C) to reach cell density around OD600 value of 0.8–1.0. The ROTOR HAD (Singer, USA) used 96-array pins to touch and transfer the yeast from liquid plate to solid plate. The 96-well format from liquid plate is pinned onto square solid plate four times (4-replicates), which becomes a 384 format. One square plate can accommodate 1536 colonies array. The colony size was scanned and quantified by “ScreenMill” for high-throughput image analysis [11]. We compared the colony size, from multiple replicates, between growth media with and without isobutanol, and also between each deletion strain and the wild-type strain. Growth abnormality caused by the presence of isobutanol alone (but not by the knockout gene of interest) was factored out through statistical analysis of wild-type colony sizes with or without isobutanol. Statistical analysis was then conducted by ScreenMill web-based program Statistics Visualization Engine (SV Engine). The *p *value of 0.05 was set as the threshold to output isobutanol-susceptible genes for further analysis.

### RNA sequencing

The yeast was incubated in 4 ml YPD at start OD_600 nm_ 0.5. The sample was treated with/without 1.2% Isobutanol and/or Trp 200 μg/ml for assay in three replicates. After 24 h, cells were harvested for total RNA extraction using QIAGEN RNeasy^®^ Mini kit (Qiagen, Germany). RNA quality was verified by Qubit™ RNA HS Assay Kit and Qubit Fluorometer (Thermo Fisher, USA). RNA Library was prepared by SuperScript™ III Reverse Transcriptase (Thermo Fisher) and TruSeq RNA LT kits (Illumina, USA) for fragment size around 350–500 bps. Sequencing was done by HiSeq 2500 with 50-bp single-end reads. Raw reads were trimmed by Trimmomatic (v0.39) and aligned to *S. cerevisiae* S288c genome (UCSC sacCer3) by TopHat. Cufflink was used to calculate FPKM values and differentially expressed genes (DEGs) were identified with the criteria of |Log2(Fold change)| >  1 and *q *value  <  0.05. Enrichment analysis of GO terms and the KEGG pathway was done by the web tool “DAVID Bioinformatics Resources 6.8” (https://david.ncifcrf.gov/tools.jsp) based on DEGs [34]. The principal components analysis (PCA) plot was processed by web tool “ClustVis” (https://biit.cs.ut.ee/clustvis/) using FPKM values as the expression levels [35]. Heatmap and hierarchical clustering were made using the R package “pheatmap (version 1.0.12)” and the web tool “Heatmapper” (www.heatmapper.ca/) [36, 37]. Clustering was based on the KEGG list enriched by the DEGs in each sample.

## Supplementary Information

**Additional file 1: Figure S1.** Growth assay of a tryptophan biosynthesis defective strain under a standard growth condition. The wild type and a tryptophan biosynthesis defective strain were tested in a standard medium with or without tryptophan addition (200 μg/ml). The growth rate shows no significantly difference between strains and between media with and without tryptophan addition. The data represent the mean ± SD (*n* = 3). **Figure S2.** Survival test of a tryptophan biosynthesis defective strain (Sc12) and a pentose phosphate pathway defective strain (Sc3). Both mutant strains Sc3 and Sc12 were sensitive to isbutanol, but could be recovered by adding tryptophan. **Figure S3.** Cell density assays of the wildtype and a tryptophan biosynthesis defective strain (Sc12) under different growth conditions. Yeasts were inoculated in 4ml medium with/without isobutanol and/or tryptophan. After 24 hours, the cell density was measured and RNA was harvested. The data represent the mean ± SD (*n* = 3). Notations: Trp, Tryptophan; IB, isobutanol. **Figure S4.** Checking the gene deletion and expression level of the mutant gene *TRP5*. **a** PCR was applied to verify the gene deletion from genome. The arrows represent the direction of 6 primers, which include A, B, C, D, kB, and kC. **b** The expression levels of the *TRP5 *gene under different growth conditions were estimated from NGS data. **Figure S5.** Expression level of gene *BNA2*. *BAN2 *is responsible for de novo biosynthesis of NAD+ from tryptophan via kynurenine pathway. It was highly expressed in WT and Sc12 under isobutanol. **Figure S6.** The recovery assay of wild type (WT) and tryptophan defective strain (Sc12) by supplementing of vitamin B1 (50 μg/ml) or tryptophan (200 μg/ml) under isobutanol (1%) stress. Under isobutanol stress, Sc12 can be only recovered by trptophan. Vitamin B1 did not help Sc12 to tolerate isobutanol. **Figure S7.** Expression level of gene *GLN3*. *GLN3 *plays a key role in yeast’s response to nitrogen starvation, including depletion of glutamine. It was upregulated when Sc12 was under isobutanol pressure; its expression level could be recovered by adding external tryptophan. **Figure S8.** Gene expression related to glutamine biosynthesis and transporter. The gene expression related to glutamine biosynthesis and transport was affected by adding tryptophan (Trp) and/or isobutanol (IB). *GLN1 *was upregluated in WT when isobutanol was added, and was upregulated in the mutant strain Sc12. *DIP5 *and *GNP1 *were up-regulated when isobutanol was added. *Avt7 *was up-regulated when WT and Sc12 were under isobutanol stress. **Figure S9.** Expression level of the *ACC1 *gene. *ACC1 *is the rate-limiting step for de novo fatty acid biosynthesis. It was up-regulated in WT under isobutanol stress. When the tryptophan pathway was defective, the expression of *ACC1 *was down-regulated under isobutanol stress but could be recovered by adding external tryptophan. **Figure S10.** Expression level of the *TAT2 *gene. *TAT2 *is a tryptophan amino acid transporter and was up-regulated when WT and Sc12 were under isobutanol stress. **Table S1.** Genes related to isobutanol tolerance. **Table S2.** Tophat mapping rate of NGS reads. **Table S3.** Gene names and numbers used in clustering analysis.

## Data Availability

The datasets supporting the conclusions of this article are included in the article and its additional files. The NGS data generated in the present study are available in the NCBI repository (GSE175794).

## Abbreviations

DEGs: Differentially expressed genes
PPP: Pentose phosphate pathway
DEF1: DNA damage-responsive RNA polymerase-degradation factor
UBC8: E2 ubiquitin-conjugating protein
GRR1: SCF ubiquitin ligase complex subunit
SLX5: SUMO-targeted ubiquitin ligase complex subunit
SLX8: SUMO-targeted ubiquitin ligase complex subunit
DOC1: Anaphase promoting complex subunit
UBP13: Ubiquitin-specific protease
UBP15: Ubiquitin-specific protease
ARO1: Pentafunctional AROM protein
ARO2: Chorismate synthase
TRP2: Anthranilate synthase
TRP4: Anthranilate phosphoribosyltransferase
TRP1: Phosphoribosylanthranilate isomerase
TRP3: Indole-3-glycerol-phosphate synthase
TRP5: Tryptophan synthase
ZWF1: Glucose-6-phosphate dehydrogenase
GND1: Phosphogluconate dehydrogenase
PRS3: Ribose phosphate diphosphokinase subunit
RPE1: Ribulose-phosphate 3-epimerase
TKL1: Transketolase
PRPP: Phosphoribosyl pyrophosphate
E4P: Erythrose 4-phosphate
SDS: Sodium dodecyl sulfate
PEP: Phosphoenolpyruvate
PCR: Polymerase chain reaction
FPKM: Fragments per kilobase of transcript per million
PCA: Principal component analysis
NAD: Nicotinamide adenine dinucleotide
Trp: Tryptophan
IB: Isobutanol
His: Histidine
Phe: Phenylalanine
Tyr: Tyrosine
Trp: Tryptophan
Val: Valine
Leu: Leucine
Ilu: Isoleucine
Cys: Cysteine
Met: Methionine
Thr: Threonine
Asp: Aspartate
Asn: Asparagine
Lys: Lysine
Glu: Glutamate
Gln: Glutamine
Arg: Arginine
SOL2: Sol2p
SOL3: 6-Phosphogluconolactonase 3
GND1: Phosphogluconate dehydrogenase
PRE1: Ribulose-phosphate 3-epimerase
PRS1: Ribose-phosphate pyrophosphokinase 1
PRS3: Ribose-phosphate pyrophosphokinase 3
PRS4: Ribose-phosphate pyrophosphokinase 4
PGM1: Phosphoglucomutase
IAA: Indole-3-acetic acid
BNA2: Tryptophan 2,3-dioxygenase
BNA7: Formylkynurenine formamidase
BNA5: Kynureninase
IDO: Indoleamine 2,3-dioxygenase
NMA2: Nicotinic acid mononucleotide adenylyltransferase
QNS1: Glutamine-dependent NAD synthetase
PN: Pyridoxine
PL: Pyridoxal
PM: Pyridoxamine
PNP: Pyridoxine 5′-phosphate
PMP: Pyridoxamine 5′-phosphate
PLP: Pyridoxal 5′-phosphate
GLN1: Glutamine synthetase
Bna5: Kynureninase
TDP: Thiamine diphosphate
ACC1: Acetyl-coA carboxylase
GLN1: Glutamine synthetase
Avt7: Vacuolar glutamine transporter
GNP1: Glutamine permease
DIP5: Dicarboxylic amino acid permease
