# Supplementary material for: Tryptophan plays an important role in yeast’s tolerance to isobutanol
Source: Biotechnol Biofuels. 2021 Oct 13;14:200. doi: 10.1186/s13068-021-02048-z (PMC8513309; doi:10.1186/s13068-021-02048-z)
Supplement: Supplementary file 1 — Additional file 1: Figure S1. Growth assay of a tryptophan biosynthesis defective strain under a standard growth condition. The wild type and a tryptophan biosynthesis defective strain were tested in a standard medium with or without tryptophan addition (200 μg/ml). The growth rate shows no significantly difference between strains and between media with and without tryptophan addition. The data represent the mean ± SD (n = 3). Figure S2. Survival test of a tryptophan biosynthesis defective strain (Sc12) and a pentose phosphate pathway defective strain (Sc3). Both mutant strains Sc3 and Sc12 were sensitive to isbutanol, but could be recovered by adding tryptophan. Figure S3. Cell density assays of the wildtype and a tryptophan biosynthesis defective strain (Sc12) under different growth conditions. Yeasts were inoculated in 4ml medium with/without isobutanol and/or tryptophan. After 24 hours, the cell density was measured and RNA was harvested. The data represent the mean ± SD (n = 3). Notations: Trp, Tryptophan; IB, isobutanol. Figure S4. Checking the gene deletion and expression level of the mutant gene TRP5. a PCR was applied to verify the gene deletion from genome. The arrows represent the direction of 6 primers, which include A, B, C, D, kB, and kC. b The expression levels of the TRP5 gene under different growth conditions were estimated from NGS data. Figure S5. Expression level of gene BNA2. BAN2 is responsible for de novo biosynthesis of NAD+ from tryptophan via kynurenine pathway. It was highly expressed in WT and Sc12 under isobutanol. Figure S6. The recovery assay of wild type (WT) and tryptophan defective strain (Sc12) by supplementing of vitamin B1 (50 μg/ml) or tryptophan (200 μg/ml) under isobutanol (1%) stress. Under isobutanol stress, Sc12 can be only recovered by trptophan. Vitamin B1 did not help Sc12 to tolerate isobutanol. Figure S7. Expression level of gene GLN3. GLN3 plays a key role in yeast’s response to nitrogen starvation, including depletio [file 13068_2021_2048_MOESM1_ESM.pdf]

Additional file 1 (Supplemental files)

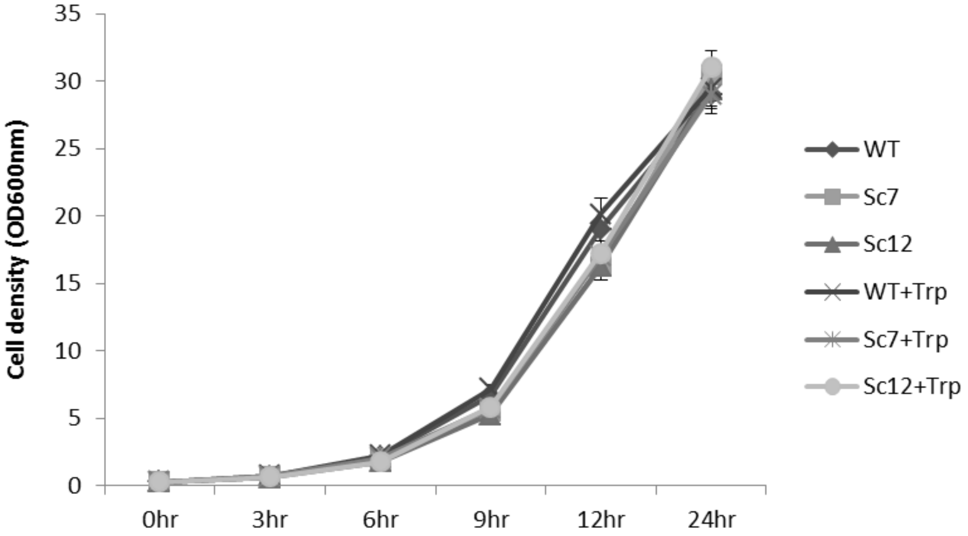

Supplemental Figure 1, Growth assay of a tryptophan biosynthesis defective strain under a standard growth condition.

The wild type and a tryptophan biosynthesis defective strain were tested in a standard medium with or without tryptophan addition (200 ug/ml). The growth rate shows no significantly difference between strains and between media with and without tryptophan addition. The data represent the mean±SD (n=3).

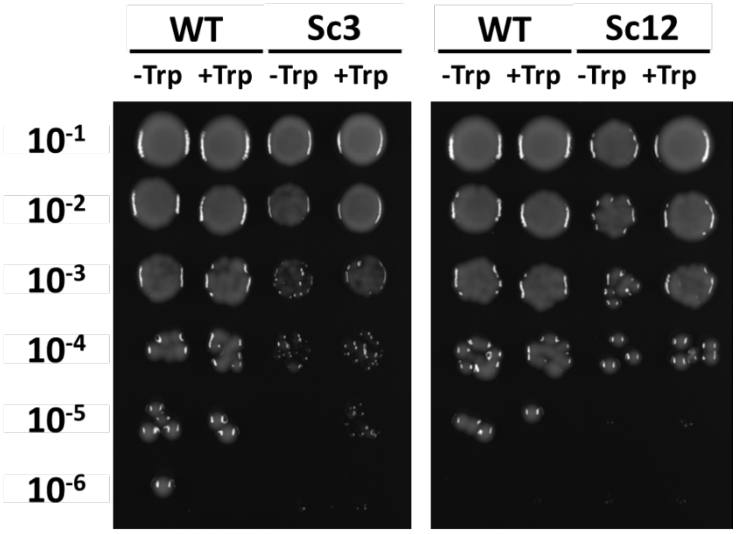

Supplemental Figure 2, Survival test of a tryptophan biosynthesis defective strain (Sc12) and a pentose phosphate pathway defective strain (Sc3)

Both mutant strains Sc3 and Sc12 were sensitive to isbutanol, but could be recovered by adding tryptophan.

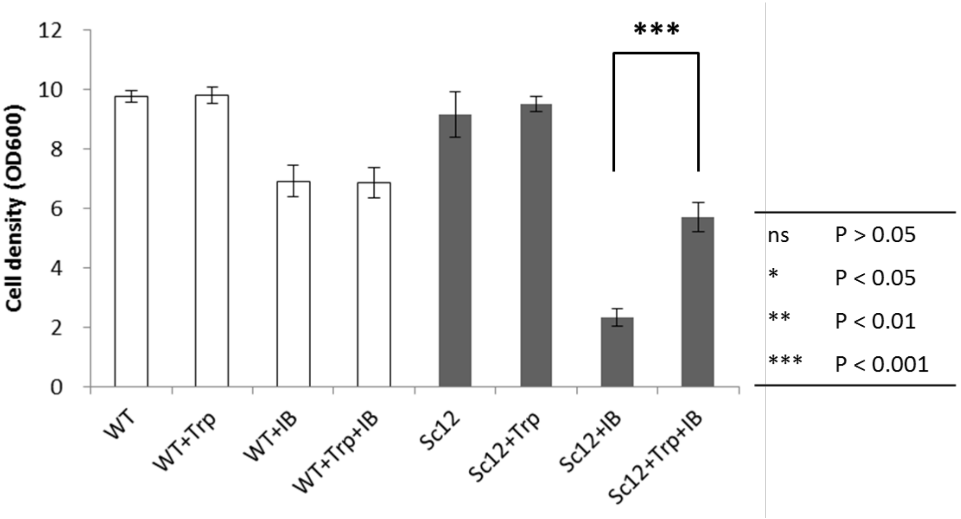

Supplemental Figure 3, Cell density assays of the wildtype and a tryptophan biosynthesis defective strain (Sc12) under different growth conditions

Yeasts were inoculated in 4ml medium with/without isobutanol and/or tryptophan. After 24 hours, the cell density was measured and RNA was harvested. The data represent the mean±SD (n=3). Notations: Trp, Tryptophan; IB, isobutanol

(a)

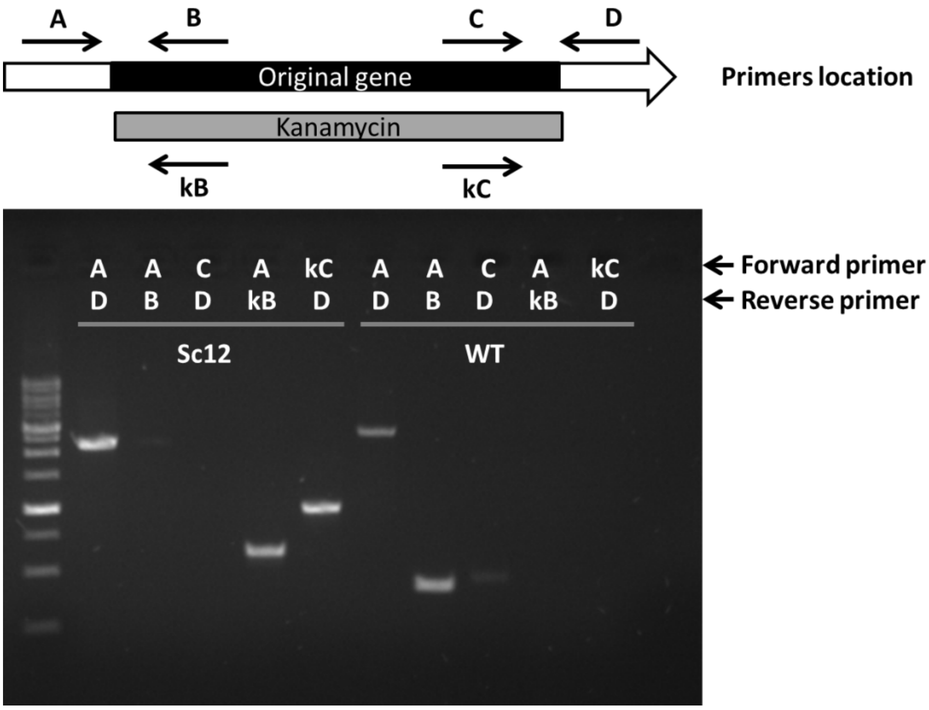

(b)

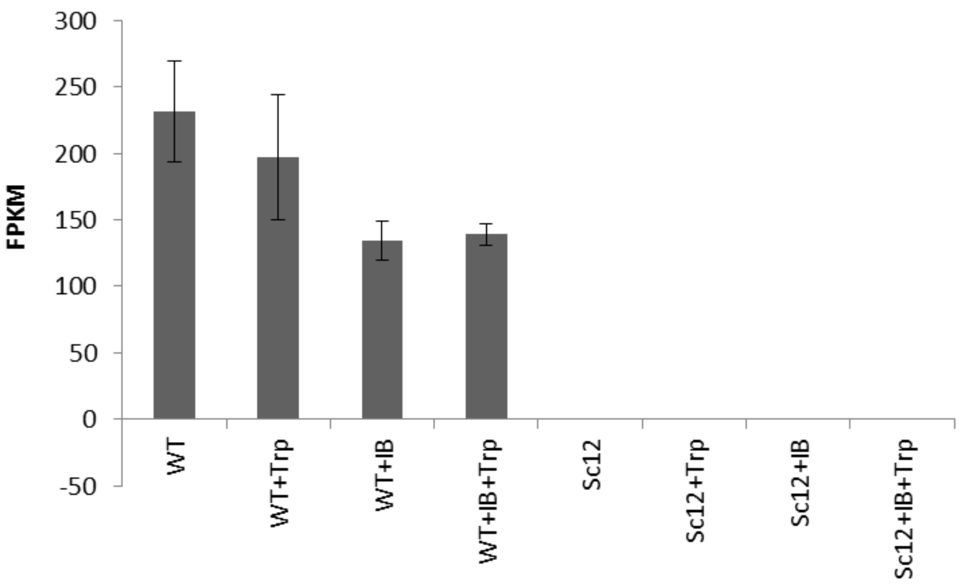

Supplemental Figure 4, Checking the gene deletion and expression level of the mutant gene *TRP5*

(a) PCR was applied to verify the gene deletion from genome. The arrows represent the direction of 6 primers, which include A, B, C, D, kB, and kC. **(b)** The expression levels of the *TRP5* gene under different growth conditions were estimated from NGS data.

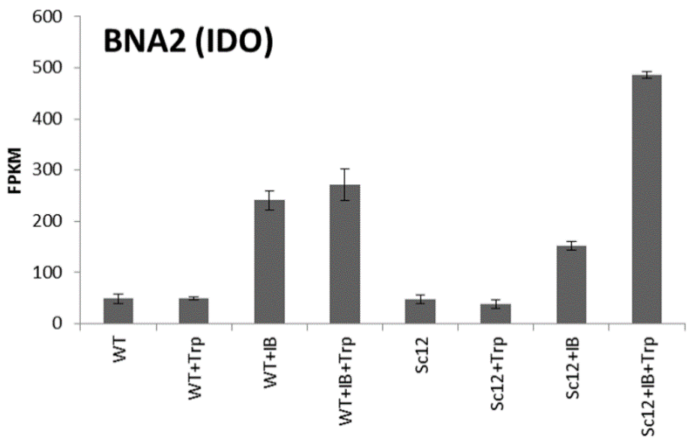

Supplemental Figure 5, Expression level of gene *BNA2*

BAN2 is responsible for de novo biosynthesis of NAD+ from tryptophan via kynurenine pathway. It was highly expressed in WT and Sc12 under isobutanol.

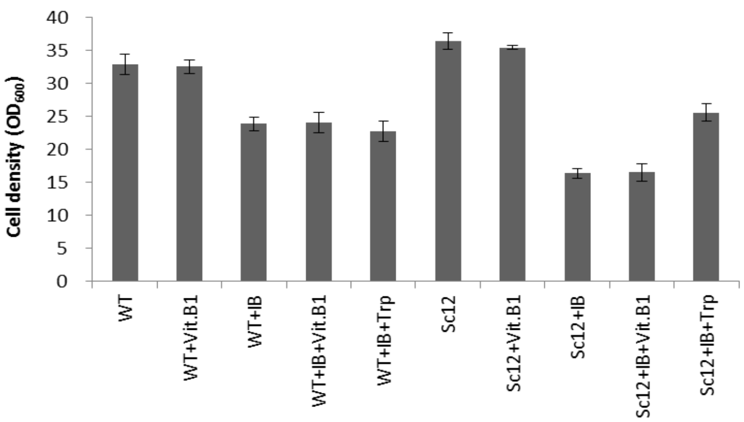

Supplemental Figure 6, **The recovery assay of wild type (WT) and tryptophan defective strain (Sc12) by supplementing of vitamin B1 (50μg/ml) or tryptophan (200μg/ml) under isobutanol (1%) stress.** Under isobutanol stress, Sc12 can be only recovered by trptophan. Vitamin B1 did not help Sc12 to tolerate isobutanol.

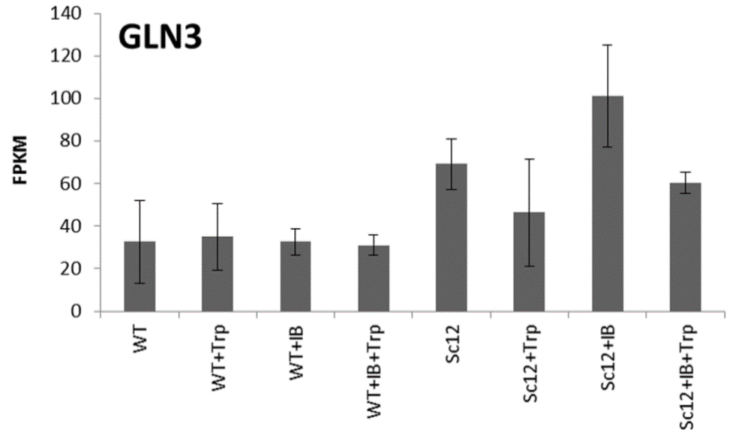

Supplemental Figure 7, Expression level of gene *GLN3*

GLN3 plays a key role in yeast's response to nitrogen starvation, including depletion of glutamine. It was upregulated when Sc12 was under isobutanol pressure; its expression level could be recovered by adding external tryptophan.

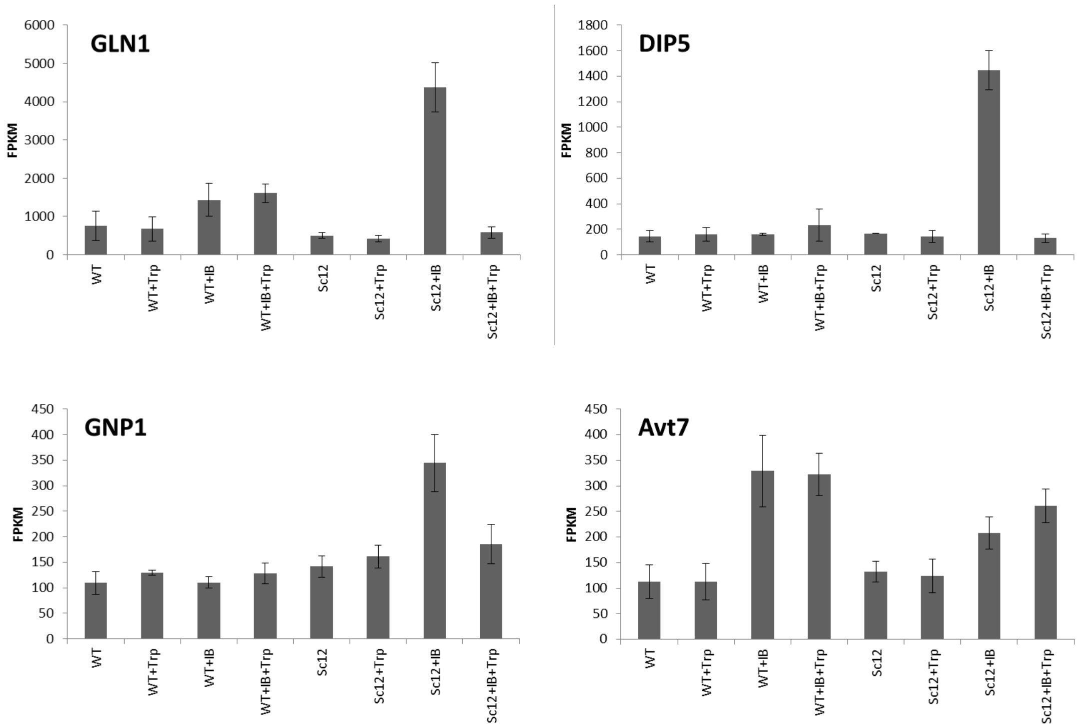

Supplemental Figure 8, Gene expression related to glutamine biosynthesis and transporter

The gene expression related to glutamine biosynthesis and transport was affected by adding tryptophan (Trp) and/or isobutanol (IB). *GLN1* was upregluated in WT when isobutanol was added, and was upregulated in the mutant strain Sc12. *DIP5* and *GNP1* were up-regulated when isobutanol was added. *Avt7* was up-regulated when WT and Sc12 were under isobutanol stress.

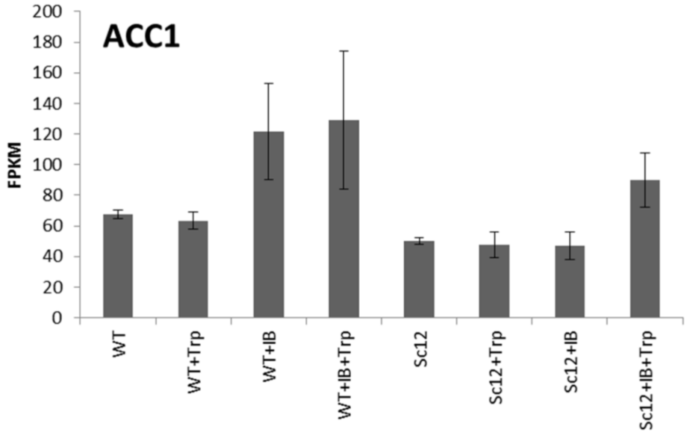

Supplemental Figure 9, Expression level of the *ACC1* gene

ACC1 is the rate-limiting step for de novo fatty acid biosynthesis. It was up-regulated in WT under isobutanol stress. When the tryptophan pathway was defective, the expression of *ACC1* was down-regulated under isobutanol stress but could be recovered by adding external tryptophan.

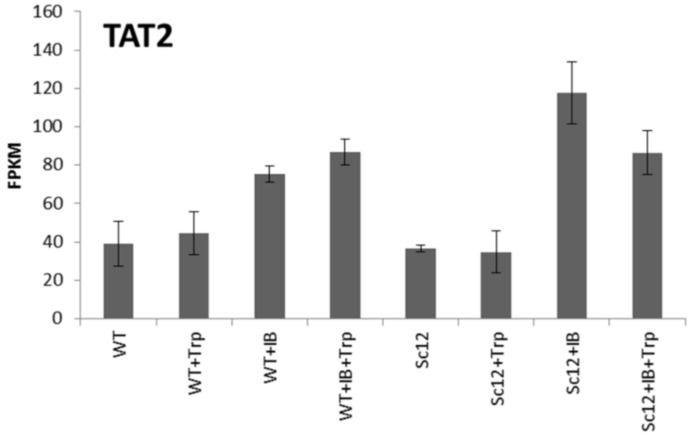

Supplemental Figure 10, Expression level of the *TAT2* gene

TAT2 is a tryptophan amino acid transporter and was up-regulated when WT and Sc12 were under isobutanol stress.

Supplemental Table 1, Genes related to isobutanol tolerance

No.	Gene	Symbol	Calculated log ratio	p-value	Annotation cluster of enrichment analysis
1	YDR127W	*ARO1*	2.074	2.83E-16	Trp biosynth
2	YKL211C	*TRP3*	1.799	7.99E-16	Trp biosynth
3	YDL225W	*SHS1*	1.864	2.65E-11	
4	YOR295W	*UAF30*	1.802	5.42E-11	
5	YER090W	*TRP2*	2.260	1.41E-10	Trp biosynth
6	YDR354W	*TRP4*	1.615	2.27E-10	Trp biosynth
7	YIL076W	*SEC28*	1.393	5.42E-10	
8	YDR007W	*TRP1*	2.216	6.06E-10	Trp biosynth
9	YPR123C		2.089	1.74E-09	
10	YNL079C	*TPM1*	2.050	1.13E-08	
11	YDL020C	*RPN4*	1.632	1.21E-08	
12	YLR110C	*CCW12*	1.597	2.50E-08	
13	YBR200W	*BEM1*	1.406	3.72E-08	
14	YJL120W		1.944	4.27E-08	
15	YOR198C	*BFR1*	1.842	5.22E-08	
16	YNL111C	*CYB5*	1.557	5.70E-08	
17	YPL188W	*POS5*	1.379	6.88E-08	
18	YFL023W	*BUD27*	1.208	7.91E-08	
19	YDR293C	*SSD1*	1.205	8.61E-08	
20	YER111C	*SWI4*	1.800	9.40E-08	
21	YJL121C	*RPE1*	1.871	1.41E-07	PPP
22	YPR153W	*44340*	1.740	5.66E-07	
23	YPR067W	*ISA2*	1.958	8.76E-07	
24	YLR044C	*PDC1*	1.018	1.00E-06	
25	YHL025W	*SNF6*	1.733	1.20E-06	
26	YAL013W	*DEP1*	1.010	1.24E-06	
27	YKL048C	*ELM1*	1.642	1.30E-06	
28	YGL026C	*TRP5*	1.669	1.64E-06	Trp biosynth
29	YDL045W-A	*MRP10*	1.360	2.49E-06	
30	YNL241C	*ZWF1*	1.637	2.59E-06	PPP
31	YBL094C		1.632	2.76E-06	
32	YMR304W	*UBP15*	1.397	4.13E-06	Ubiquitination
33	YGL168W	*HUR1*	1.545	5.16E-06	
34	YDR207C	*UME6*	1.722	6.33E-06	

35	YDL172C		1.267	7.24E-06	
36	YLR087C	*CSF1*	0.935	7.61E-06	
37	YCR081W	*SRB8*	1.263	7.74E-06	
38	YGL148W	*ARO2*	1.516	7.78E-06	Trp biosynth
39	YER083C	*GET2*	1.254	8.21E-06	
40	YBR171W	*SEC66*	1.280	9.83E-06	
41	YCL046W		1.238	1.09E-05	
42	YLR386W	*VAC14*	1.682	1.15E-05	
43	YCL045C	*EMC1*	1.225	1.36E-05	
44	YNL066W	*SUN4*	1.578	1.38E-05	
45	YJR090C	*GRR1*	1.733	1.59E-05	Ubiquitination
46	YLR342W	*FKS1*	1.659	1.61E-05	
47	YLR111W		0.902	1.63E-05	
48	YNL133C	*FYV6*	1.558	1.80E-05	
49	YDR186C	*SND1*	1.644	1.99E-05	
50	YCL037C	*SRO9*	1.193	2.29E-05	
51	YJL183W	*MNN11*	1.623	2.70E-05	
52	YHR183W	*GND1*	1.178	2.92E-05	PPP
53	YDL052C	*SLC1*	1.194	4.04E-05	
54	YPR139C	*LOA1*	1.426	4.40E-05	
55	YBR095C	*RXT2*	1.177	5.22E-05	
56	YMR032W	*HOF1*	0.845	5.61E-05	
57	YAL021C	*CCR4*	1.621	5.86E-05	
58	YDL018C	*ERP3*	1.167	6.14E-05	
59	YPR074C	*TKL1*	1.437	6.93E-05	PPP
60	YDR008C		1.112	8.87E-05	
61	YNR021W		1.420	9.95E-05	
62	YHR008C	*SOD2*	1.393	1.20E-04	
63	YGL020C	*GET1*	1.532	1.58E-04	
64	YHR060W	*VMA22*	1.065	1.65E-04	
65	YBR106W	*SND3*	1.085	2.08E-04	
66	YER122C	*GLO3*	1.493	2.36E-04	
67	YBR134W		1.060	2.95E-04	
68	YHL011C	*PRS3*	1.297	3.64E-04	PPP
69	YMR007W		0.748	3.89E-04	
70	YNL064C	*YDJ1*	1.292	4.29E-04	
71	YIL052C	*RPL34B*	0.801	4.34E-04	

72	YER141W	*COX15*	1.201	4.45E-04	
73	YDR364C	*CDC40*	0.899	5.18E-04	
74	YBR111W-A	*SUS1*	1.405	5.84E-04	
75	YHR180W		1.249	6.21E-04	
76	YNL315C	*ATP11*	1.376	7.70E-04	
77	YCL007C		1.370	8.15E-04	
78	YGR104C	*SRB5*	1.143	8.45E-04	
79	YKL098W	*MTC2*	1.141	8.63E-04	
80	YDR418W	*RPL12B*	0.863	8.81E-04	
81	YDR245W	*MNN10*	1.362	8.87E-04	
82	YLR224W	*UCC1*	1.133	9.35E-04	Ubiquitination
83	YDR204W	*COQ4*	1.337	1.05E-03	
84	YFL001W	*DEG1*	1.115	1.16E-03	
85	YOR200W		1.102	1.30E-03	
86	YBR097W	*VPS15*	1.319	1.32E-03	
87	YMR326C		0.917	1.34E-03	
88	YOR044W	*IRC23*	1.134	1.40E-03	
89	YPR132W	*RPS23B*	1.117	1.46E-03	
90	YJL006C	*CTK2*	1.307	1.48E-03	
91	YEL012W	*UBC8*	1.165	1.51E-03	Ubiquitination
92	YMR183C	*SSO2*	0.671	1.57E-03	
93	YKL139W	*CTK1*	1.161	1.67E-03	
94	YML011C	*RAD33*	0.663	1.78E-03	
95	YJR066W	*TOR1*	1.058	2.09E-03	
96	YBR133C	*HSL7*	1.246	2.56E-03	
97	YLR207W	*HRD3*	1.025	2.86E-03	
98	YHR013C	*ARD1*	0.843	3.07E-03	
99	YNR052C	*POP2*	0.878	3.10E-03	
100	YER066C-A		0.826	3.78E-03	
101	YJL184W	*GON7*	1.196	3.96E-03	
102	YKL011C	*CCE1*	0.989	4.04E-03	
103	YBR144C		0.855	4.07E-03	
104	YJL176C	*SWI3*	1.201	4.52E-03	
105	YFR019W	*FAB1*	0.837	4.97E-03	
106	YGL167C	*PMR1*	0.962	5.36E-03	
107	YKL054C	*DEF1*	0.950	5.83E-03	Ubiquitination
108	YFR033C	*QCR6*	0.969	5.96E-03	

109	YGL024W		0.968	5.98E-03	
110	YLR233C	*EST1*	1.164	6.52E-03	
111	YPR089W		1.012	6.53E-03	
112	YDL185W	*VMA1*	1.135	6.57E-03	
113	YER042W	*MXR1*	0.768	7.22E-03	
114	YGL240W	*DOC1*	1.122	7.28E-03	Ubiquitination
115	YDR532C	*KRE28*	0.803	7.30E-03	
116	YJR011C		0.773	7.32E-03	
117	YCR045C	*RRT12*	0.769	7.61E-03	
118	YGR208W	*SER2*	0.761	7.79E-03	
119	YAL016W	*TPD3*	1.108	8.15E-03	
120	YJR075W	*HOC1*	0.984	8.33E-03	
121	YOR331C		1.102	8.52E-03	
122	YBR077C	*SLM4*	0.983	8.58E-03	
123	YGR272C		1.095	9.01E-03	
124	YGL023C	*PIB2*	0.912	9.77E-03	
125	YCL062W		0.739	9.92E-03	
126	YLR292C	*SEC72*	1.120	9.96E-03	
127	YHR135C	*YCK1*	0.738	9.98E-03	
128	YBL100C		0.962	1.02E-02	
129	YCL042W		0.729	1.10E-02	
130	YFL003C	*MSH4*	0.881	1.12E-02	
131	YER019C-A	*SBH2*	0.726	1.14E-02	
132	YEL036C	*ANP1*	1.062	1.16E-02	
133	YGL192W	*IME4*	1.054	1.24E-02	
134	YDR363W-A	*SEM1*	0.877	1.30E-02	
135	YPL045W	*VPS16*	1.039	1.38E-02	
136	YCL061C	*MRC1*	0.703	1.44E-02	
137	YAL047C	*SPC72*	0.694	1.67E-02	
138	YLL027W	*ISA1*	1.011	1.70E-02	
139	YDL013W	*SLX5*	0.716	1.79E-02	Ubiquitination
140	YER014C-A	*BUD25*	0.998	1.86E-02	
141	YJR105W	*ADO1*	0.887	1.88E-02	
142	YLR093C	*NYV1*	0.504	1.95E-02	
143	YFR032C-A	*RPL29*	0.808	2.26E-02	
144	YBL067C	*UBP13*	0.801	2.38E-02	Ubiquitination
145	YNL098C	*RAS2*	0.853	2.41E-02	

146	YER078C	*ICP55*	0.829	2.93E-02	
147	YGL180W	*ATG1*	0.760	3.01E-02	
148	YDR417C		0.926	3.08E-02	
149	YJL140W	*RPB4*	0.984	3.24E-02	
150	YBR196C-A		0.917	3.27E-02	
151	YDL047W	*SIT4*	0.745	3.37E-02	
152	YKL096W-A	*CWP2*	0.805	3.43E-02	
153	YGR105W	*VMA21*	0.731	3.58E-02	
154	YGR210C		0.785	4.06E-02	
155	YDR226W	*ADK1*	0.954	4.12E-02	
156	YER116C	*SLX8*	0.716	4.18E-02	Ubiquitination
157	YML035C	*AMD1*	0.773	4.30E-02	
158	YPR135W	*CTF4*	0.716	4.43E-02	
159	YNL025C	*SSN8*	0.764	4.58E-02	
160	YML124C	*TUB3*	0.767	4.61E-02	
161	YBR173C	*UMP1*	0.695	4.87E-02	Ubiquitination

Abbreviation: Isobutanol, IB; Trp, Tryptophan; PPP, Pentose phosphate pathway

Supplemental Table 2, Tophat mapping rate of NGS reads

Samples	Input	Mapped	Mapped%
1. WT	18,220,832	17,985,218	98.7%
2. WT	18,534,880	18,310,476	98.8%
3. WT	18,264,239	18,021,675	98.7%
4. WT+Trp	19,048,752	18,715,890	98.3%
5. WT+Trp	17,865,622	17,635,022	98.7%
6. WT+Trp	17,557,389	16,990,440	96.8%
7. WT+IB	18,683,975	18,371,614	98.3%
8. WT+IB	17,800,152	17,515,351	98.4%
9. WT+IB	13,837,961	13,610,922	98.4%
10. WT+Trp+IB	14,772,542	14,532,935	98.4%
11. WT+Trp+IB	14,674,845	14,428,832	98.3%
12. WT+Trp+IB	13,843,855	13,631,284	98.5%
13. Sc12	13,602,232	13,407,182	98.6%
14. Sc12	13,505,045	13,350,107	98.9%
15. Sc12	8,618,503	8,506,648	98.7%
16. Sc12+Trp	9,197,535	9,073,303	98.6%
17. Sc12+Trp	15,935,132	15,720,327	98.7%
18. Sc12+Trp	15,186,545	14,986,752	98.7%
19. Sc12+IB	15,868,238	15,668,376	98.7%
20. Sc12+IB	14,699,642	14,516,412	98.8%
21. Sc12+IB	14,990,531	14,853,447	99.1%
22. Sc12+Trp+IB	14,345,141	14,122,303	98.4%
23. Sc12+Trp+IB	13,622,548	13,423,455	98.5%
24. Sc12+Trp+IB	14,857,726	14,644,703	98.6%

Abbreviation: Trp = Tryptophan, IB = Isobutanol

Supplemental Table 3, Gene names and numbers used in clustering analysis

1. Biosynthesis of amino acids (Figure 5a)

No.	Genes	Gene synonyms
**1**	YAL012W	*CYS3, CYI1, FUN35, STR1*
**2**	YAL038W	*CDC19, PYK1*
**3**	YBL068W	*PRS4*
**4**	YBR115C	*LYS2*
**5**	YBR117C	*TKL2*
**6**	YBR166C	*TYR1*
**7**	YBR218C	*PYC2*
**8**	YBR248C	*HIS7*
**9**	YBR249C	*ARO4*
**10**	YBR263W	*SHM1, SHMT1, TMP3*
**11**	YCL009C	*ILV6*
**12**	YCL030C	*HIS4*
**13**	YCL064C	*CHA1*
**14**	YCR005C	*CIT2*
**15**	YCR012W	*PGK1*
**16**	YCR053W	*THR4*
**17**	YDL021W	*GPM2*
**18**	YDL066W	*IDP1*
**19**	YDL131W	*LYS21*
**20**	YDL171C	*GLT1*
**21**	YDL182W	*LYS20*
**22**	YDR007W	*TRP1*
**23**	YDR035W	*ARO3*
**24**	YDR050C	*TPI1*
**25**	YDR111C	*ALT2*
**26**	YDR127W	*ARO1*
**27**	YDR158W	*HOM2, THR2*
**28**	YDR234W	*LYS4, LYS3*
**29**	YDR300C	*PRO1*
**30**	YDR354W	*TRP4*
**31**	YDR502C	*SAM2, ETH2*
**32**	YEL046C	*GLY1*
**33**	YER023W	*PRO3, ORE2*
**34**	YER052C	*HOM3, BOR1, SIL4, THR3*

**35**	YER055C	*HIS1*
**36**	YER069W	*ARG5,6*
**37**	YER081W	*SER3*
**38**	YER086W	*ILV1, ISO1*
**39**	YER090W	*TRP2*
**40**	YER091C	*MET6*
**41**	YER099C	*PRS2*
**42**	YFR025C	*HIS2*
**43**	YFR055W	*IRC7*
**44**	YGL009C	*LEU1*
**45**	YGL026C	*TRP5*
**46**	YGL062W	*PYC1*
**47**	YGL148W	*ARO2*
**48**	YGL184C	*STR3*
**49**	YGL202W	*ARO8*
**50**	YGR012W	*MCY1*
**51**	YGR043C	*NQM1*
**52**	YGR124W	*ASN2*
**53**	YGR155W	*CYS4, NHS5, STR4, VMA41*
**54**	YGR192C	*TDH3, GLD1, HSP35, HSP36, S*
**55**	YGR208W	*SER2*
**56**	YGR240C	*PFK1*
**57**	YGR254W	*ENO1, HSP48*
**58**	YHL011C	*PRS3*
**59**	YHR018C	*ARG4*
**60**	YHR025W	*THR1*
**61**	YHR033W	Uncharacterized
**62**	YHR174W	*ENO2*
**63**	YHR208W	*BAT1, ECA39, TWT1*
**64**	YIL020C	*HIS6*
**65**	YIL074C	*SER33*
**66**	YIL094C	*LYS12, LYS10, LYS11*
**67**	YIL116W	*HIS5*
**68**	YIR034C	*LYS1*
**69**	YJL052W	*TDH1, GLD3*
**70**	YJL071W	*ARG2, HRB574*
**71**	YJL088W	*ARG3*
**72**	YJL121C	*RPE1, EPI1, POS18*

**73**	YJL200C	*ACO2*
**74**	YJR009C	*TDH2, GLD2*
**75**	YJR016C	*ILV3*
**76**	YJR130C	*STR2*
**77**	YJR139C	*HOM6, THR6*
**78**	YJR148W	*BAT2, ECA40, TWT2*
**79**	YKL060C	*FBA1, LOT1*
**80**	YKL106W	*AAT1*
**81**	YKL152C	*GPM1*
**82**	YKL181W	*PRS1, PRP1*
**83**	YKL211C	*TRP3*
**84**	YLL058W	Putative cystathionine gamma-synthase
**85**	YLR027C	*AAT2, ASP5*
**86**	YLR058C	*SHM2, SHMT2*
**87**	YLR089C	*ALT1*
**88**	YLR174W	*IDP2*
**89**	YLR180W	*SAM1, ETH10*
**90**	YLR304C	*ACO1, GLU1*
**91**	YLR354C	*TAL1*
**92**	YLR355C	*ILV5*
**93**	YML082W	Putative carbon-sulfur lyase
**94**	YMR062C	*ARG7*
**95**	YMR108W	*ILV2, SMR1, THI1*
**96**	YMR205C	*PFK2*
**97**	YMR323W	*ERR3*
**98**	YNL009W	*IDP3*
**99**	YNL037C	*IDH1*
**100**	YNL104C	*LEU4*
**101**	YNL277W	*MET2*
**102**	YNL316C	*PHA2*
**103**	YNR001C	*CIT1, LYS6*
**104**	YNR050C	*LYS9, LYS13*
**105**	YOL056W	*GPM3*
**106**	YOL058W	*ARG1, ARG10*
**107**	YOL061W	*PRS5*
**108**	YOL140W	*ARG8*
**109**	YOR095C	*RKI1*
**110**	YOR108W	*LEU9*

**111**	YOR136W	*IDH2*
**112**	YOR184W	*SER1, ADE9*
**113**	YOR202W	*HIS3, HIS10, HIS8*
**114**	YOR323C	*PRO2*
**115**	YOR347C	*PYK2*
**116**	YPL111W	*CAR1, LPH15*
**117**	YPR001W	*CIT3*
**118**	YPR035W	*GLN1*
**119**	YPR060C	*ARO7, HGS1, OSM2, TYR7*
**120**	YPR074C	*TKL1*
**121**	YPR145W	*ASN1*

2. Amino acid transporters (Figure 5b)

**No.**	**Genes**	**Gene Synonyms**	**Transport direction**	**Substrate(s)**
**1**	YCL025C	*Agp1*	ext to cyt	Broad range (not Lys or Arg); Ile, Leu, Phe, Cys, Gln, Asn
**2**	YBR132C	*Agp2*	ext to cyt	Val, Ile, Leu, Phe, Thr
**3**	YFL055W	*Agp3*	ext to cyt	Val, Ile, Leu, Phe, Ser, Thr, Glu, Asp
**4**	YNL270C	*Alp1*	ext to cyt	Arg
**5**	YBR068C	*Bap2*	ext to cyt	Val, Ile, Leu, Ala, Phe, Tyr, Met, Cy
**6**	YDR046C	*Bap3*	ext to cyt	Val, Ile, Leu, Ala, Phe, Tyr, Trp, Met, Cys, Thr
**7**	YEL063C	*Can1*	ext to cyt	His, Lys, Arg, Orn
**8**	YPL265W	*Dip5*	ext to cyt	Ala, Gly, Ser, Gln, Asn, Glu, Asp
**9**	YKR039W	*Gap1*	ext to cyt	All natural aa, Cit, Orn, Leu
**10**	YDR508C	*Gnp1*	ext to cyt	Leu, Pro, Met, Cys, Ser, Thr, Gln, Asn
**11**	YGR191W	*Hip1*	ext to cyt	His
**12**	YLL061W	*Mmp1*	ext to cyt	S-Methylmethionine
**13**	YNL268W	*Lyp1*	ext to cyt	Met, Lys
**14**	YOR348C	*Put4*	ext to cyt	Ala, Gly, Pro
**15**	YPL274W	*Sam3*	ext to cyt	S-Adenosylmethionine
**16**	YBR069C	*Tat1*	ext to cyt	Val, Leu, Ile, Cys, Thr, Tyr, Trp, His
**17**	YOL020W	*Tat2*	ext to cyt	Ala, Gly, Cys, Phe, Tyr, Trp
**18**	YGR055W	*Mup1*	ext to cyt	Met, Cys
**19**	YHL036W	*Mup3*	ext to cyt	Met
**20**	YLL055W	*Yct1*	ext to cyt	Cys
**21**	YKR105C	*Vba5*	cyt to ext	Lys, Arg

**22**	YMR088C	*Vba1*	cyt to vac	His, Lys
**23**	YBR293W	*Vba2*	cyt to vac	His, Lys, Arg
**24**	YCL069W	*Vba3*	cyt to vac	His, Lys
**25**	YCL038C	*Atg22*	vact to cy	Ile, Leu, Tyr
**26**	YJR001W	*Avt1*	cyt to vac	Neutral aa, His
**27**	YKL146W	*Avt3*	vact to cy	Neutral aa
**28**	YNL101W	*Avt4*	vact to cy	Neutral aa, His, Lys, Arg
**29**	YER119C	*Avt6*	vact to cy	Glu, Asp
**30**	YIL088C	*Avt7*	vact to cy	Pro, Gln
**31**	YCR075C	*Ers1*	vact to cy	Cys
**32**	YOL092W	*Ypq1*	cyt to vac	Lys, Arg
**33**	YDR352W	*Ypq2*	cyt to vac	Arg
**34**	YBR147W	*Ypq3*	cyt to vac	His
**35**	YPR021C	*Agc1*	cyt ↔ mit	Glu, Asp
**36**	YDL119C	*Hem25*	cyt ↔ mit	Gly
**37**	YOR130C	*Ort1*	cyt ↔ mit	Lys, Arg, Orn
**38**	YNL003C	*Sam5*	cyt ↔ mit	S-Adenosylmethionine, S-adenosylhomocysteine
**39**	YDL210W	*Uga4*	ext to cyt or cyt to vac	γ -Aminobutyric acid
**40**	YNL065W	*Aqr1*	cyt to ext	Ala, Glu, Asp

Abbreviation: ext: extracellular; cyt: cytosol; vac: vacuolar lumen; mit: mitochondrial matrix; aa: amino acids. Reference: Microbiology and Molecular Biology Reviews 83.4 (2019): e00024-19.

3. Pentose phosphate pathway (Figure 5c)

**No.**	**Genes**	**Gene Synonyms**
**1**	YBL068W	PRS4
**2**	YBR117C	TKL2
**3**	YBR196C	PGI1, CDC30
**4**	YCR036W	RBK1
**5**	YCR073W-A	SOL2, YCRX13W
**6**	YDR248C	Putative gluconokinase
**7**	YER099C	PRS2
**8**	YGR043C	NQM1
**9**	YGR240C	PFK1
**10**	YGR248W	SOL4

**11**	YGR256W	GND2
**12**	YHL011C	PRS3
**13**	YHR163W	SOL3
**14**	YHR183W	GND1
**15**	YJL121C	RPE1, EPI1, POS18
**16**	YKL060C	FBA1, LOT1
**17**	YKL127W	PGM1
**18**	YKL181W	PRS1, PRP1
**19**	YLR354C	TAL1
**20**	YLR377C	FBP1, ACN8
**21**	YMR105C	PGM2, GAL5
**22**	YMR205C	PFK2
**23**	YMR278W	PRM15, PGM3
**24**	YNL241C	ZWF1, MET19, POS10
**25**	YNR034W	SOL1
**26**	YOL061W	PRS5
**27**	YOR095C	RKI1
**28**	YPR074C	TKL1

4. Tryptophan metabolism (Figure 5d)

No.	Genes	Gene Synonyms
**1**	YJR078W	*BNA2*
**2**	YDR428C	*BNA7*
**3**	YBL098W	*BNA4*
**4**	YLR231C	*BNA5*
**5**	YJR025C	*BNA1*
**6**	YDR148C	*KGD2*
**7**	YFL018C	*LPD1*
**8**	YPL028W	*ERG10*
**9**	YJL060W	*BNA3*
**10**	YOR374W	*ALD4*
**11**	YPL061W	*ALD6*
**12**	YER073W	*ALD5*
**13**	YMR110C	*HFD1*
**14**	YMR169C	*ALD3*
**15**	YMR170C	*ALD2*
**16**	YGL202W	*ARO8*
**17**	YIL164C	*NIT1*

**18**	YDR242W	*AMD2*
**19**	YDR256C	*CTA1*
**20**	YGR088W	*CTT1*

5. Nicotinate and nicotinamide metabolism (Figure 5e)

No.	Genes	Gene Synonyms
**1**	YFR047C	*BNA6*
**2**	YOR209C	*NPT1*
**3**	YLR209C	*PNP1*
**4**	YNL129W	*NRK1*
**5**	YOR155C	*ISN1*
**6**	YGL224C	*SDT1*
**7**	YDR400W	*URH1*
**8**	YLR328W	*NMA1*
**9**	YGR010W	*NMA2*
**10**	YCL047C	*POF1*
**11**	YGL067W	*NPY1*
**12**	YHR074W	*QNS1*
**13**	YGL037C	*PNC1*
**14**	YDL042C	*SIR2*
**15**	YOL068C	*HST1*
**16**	YPL015C	*HST2*
**17**	YDR191W	*HST4*
**18**	YOR025W	*HST3*
**19**	YJR049C	*UTR1*
**20**	YBR006W	*UGA2*

6. Vitamin B6 metabolism (Figure 5f)

No.	Genes	Gene Synonyms
**1**	YBR035C	*PDX3*
**2**	YCR053W	*THR4*
**3**	YEL029C	*BUD16*
**4**	YFL059W	*SNZ3*
**5**	YFL060C	*SNO3*
**6**	YGR017W	Uncharacterized protein
**7**	YMR095C	*SNO1*
**8**	YMR096W	*SNZ1*
**9**	YNL333W	*SNZ2*

**10**	YNL334C	*SNO2*
**11**	YNR027W	*BUD17*
**12**	YOR184W	*SER1, ADE9*
**13**	YPR127W	Putative pyridoxine 4-dehydrogenase

7. Thiamine metabolism: Vitamin B_1 (Figure 5g)

**No.**	**Genes**	**Gene Synonyms**
**1**	YAR071W	*PHO11*
**2**	YBR092C	*PHO3*
**3**	YBR093C	*PHO5*
**4**	YCL017C	*NFS1, SPL1*
**5**	YDL024C	*DIA3*
**6**	YDL244W	*THI13*
**7**	YDR226W	*ADK1, AKY1, AKY2*
**8**	YDR481C	*PHO8*
**9**	YER170W	*ADK2, AKY3, PAK3*
**10**	YFL058W	*THI5*
**11**	YGR144W	*THI4, ESP*
**12**	YHR215W	*PHO12, PHO10*
**13**	YJR156C	*THI11*
**14**	YNL332W	*THI12*
**15**	YOL055C	*THI20*
**16**	YOR143C	*THI80*
**17**	YPL214C	*THI6*
**18**	YPL258C	*THI21*
**19**	YPR073C	*LTP*

8. Fatty acid metabolism (Figure 5h)

**No.**	**Genes**	**Gene Synonyms**
**1**	YBR026C	*ETR1*
**2**	YBR159W	*IFA38*
**3**	YCR034W	*ELO2, FEN1, GNS1, VBM2*
**4**	YDL015C	*TSC13*
**5**	YER015W	*FAA2, FAM1*
**6**	YER061C	*CEM1*
**7**	YGL055W	*OLE1, MDM2*
**8**	YGL205W	*POX1, FOX1*
**9**	YHR067W	*HTD2, RMD12*

**10**	YIL009W	*FAA3*
**11**	YIL160C	*POT1, FOX3, POX3*
**12**	YJL097W	*PHS1*
**13**	YJL196C	*ELO1*
**14**	YKL055C	*OAR1*
**15**	YKL182W	*FAS1*
**16**	YLR372W	*ELO3, APA1, SRE1, SUR4, VBM1*
**17**	YMR207C	*HFA1*
**18**	YMR246W	*FAA4*
**19**	YNR016C	*ACC1,*
**20**	YOR221C	*MCT1*
**21**	YOR317W	*FAA1*
**22**	YPL028W	*ERG10, LPB3, TSM0115*
**23**	YPL231W	*FAS2*
